# Mito-nuclear genetic comparison in a *Wolbachia *infected weevil: insights on reproductive mode, infection age and evolutionary forces shaping genetic variation

**DOI:** 10.1186/1471-2148-10-340

**Published:** 2010-11-04

**Authors:** Marcela S Rodriguero, Analía A Lanteri, Viviana A Confalonieri

**Affiliations:** 1 Departamento de Ecología, Genética y Evolución, Facultad de Ciencias Exactas y Naturales, Universidad de Buenos Aires, 1428, Ciudad Autónoma de Buenos Aires, Provincia de Buenos Aires, Argentina; 2 División Entomología, Museo de La Plata, Facultad de Ciencias Naturales y Museo, Universidad Nacional de La Plata, La Plata, 1900, Provincia de Buenos Aires, Argentina

## Abstract

**Background:**

Maternally inherited endosymbionts like *Wolbachia pipientis *are in linkage disequilibrium with the mtDNA of their hosts. Therefore, they can induce selective sweeps, decreasing genetic diversity over many generations. This sex ratio distorter, that is involved in the origin of parthenogenesis and other reproductive alterations, infects the parthenogenetic weevil *Naupactus cervinus*, a serious pest of ornamental and fruit plants.

**Results:**

Molecular evolution analyses of mitochondrial (*COI*) and nuclear (*ITS1*) sequences from 309 individuals of *Naupactus cervinus *sampled over a broad range of its geographical distribution were carried out. Our results demonstrate lack of recombination in the nuclear fragment, non-random association between nuclear and mitochondrial genomes and the consequent coevolution of both genomes, being an indirect evidence of apomixis. This weevil is infected by a single *Wolbachia *strain, which could have caused a moderate bottleneck in the invaded population which survived the initial infection.

**Conclusions:**

Clonal reproduction and *Wolbachia *infection induce the coevolution of bacterial, mitochondrial and nuclear genomes. The time elapsed since the *Wolbachia *invasion would have erased the traces of the demographic crash in the mtDNA, being the nuclear genome the only one that retained the signal of the bottleneck. The amount of genetic change accumulated in the mtDNA and the high prevalence of *Wolbachia *in all populations of *N. cervinus *agree with the hypothesis of an ancient infection. *Wolbachia *probably had great influence in shaping the genetic diversity of *N. cervinus*. However, it would have not caused the extinction of males, since sexual and asexual infected lineages coexisted until recent times.

## Background

Parthenogenetic reproduction is fairly common in Curculionidae [1,2]. Many cases have been reported in three different subfamilies: Scolytinae (bark beetles), Listroderinae and Entiminae (broad-nose weevils), especially in species from the Old World (*e.g*. [1,3-6]). Although less studied, weevils from South America also concentrate a huge number of asexual species. For instance, Lanteri and Normark [7] provided a list of more than 30 parthenogenetic or presumptive parthenogenetic species within the tribe Naupactini (subfamily Entiminae). There is no direct evidence of parthenogenesis for the majority of these species. However, Floyd F. Smith and colleagues from the United States Department of Agriculture (USDA) proved this reproductive mode through rearing experiments for the species *Naupactus cervinus *and *N. leucoloma *respectively (see [8]), and Marvaldi [9] did the same with *N. ruizi*.

Parthenogenesis in Curculionidae is thelytokous and apomictic (*i.e*. ameiotic) [2]. Apomixis was confirmed for several Old World broad-nosed weevils [3,10], although nothing is known for South American species. As a consequence of this kind of reproduction, progeny would result in a group of females genetically identical to their mothers [11]. Nevertheless, the absence of recombination would originate heterozygote genotypes, as rare mutations accumulate [12,13]. The lack of meiosis could be detrimental in the short term [14-16], although heterozygosity could explain the higher dispersion and adaptation ability of some parthenogenetic species over the sexual ones in the long term [1].

Traditionally, hybridization and polyploidy were the main traits invoked to explain the origin of asexuality in weevils [2,17]. However, the report of the parthenogenesis inductor bacterium *Wolbachia pipientis *(hereafter "*Wolbachia*") in several species of the tribe Naupactini [18,19] and also in the genera *Cathormiocerus *(Entiminae, Trachyphloeini) [20] and *Otiorhynchus *(Entiminae, Otiorhynchini) [21], suggested another possible explanation for the origin of this reproductive mode in broad-nosed weevils.

*Wolbachia *infection can produce drastic consequences on the evolution of its host species, such as extinction or sex role reversal [22,23]. However, it can also affect the population genetics and molecular evolution of the vertical transmitted genomes (mitochondrial, and for apomictic species also nuclear DNA), because of the linkage disequilibrium among these cotransmitted molecules [24-26].

When an arthropod population is invaded by a *Wolbachia *strain that rapidly spreads, the host mtDNA (and perhaps the nDNA) associated to the initial infection (*i.e*. in linkage disequilibrium with the bacterial genome) will hitchhike through the population to fixation [26,27]. Consequently, this selective sweep ("indirect selection on the mtDNA") will lead to a loss of mtDNA/nDNA diversity in part of the host species distribution. Since the expected coalescent time of mtDNA in an uninfected species is twice the effective population size of females (*N_f_*), a decrease in mtDNA/nDNA diversity will be evident for *ca*. 2*N_f _*generations after the *Wolbachia *invasion [25]. Then, current levels of genetic diversity in infected arthropod populations should be analyzed in the context of the infection age.

The spread and prevalence of the infection can also be related to other important factors. For instance, the success of the infected females in producing more daughters than uninfected females [28]. If infected females have a higher fitness than unifected ones, a perfect transmission of the endosymbiont will lead to a high prevalence or to its fixation [23].

From the considerations stated before, we formulate the following hypotheses:

i) If parthenogenesis is apomictic, the lack of meiosis precludes the occurrence of recombination;

ii) Then, asexuality can generate linkage disequilibrium among mitochondrial and nuclear genomes;

iii) Vertical transmission of *Wolbachia *can generate linkage disequilibrium among mitochondrial, nuclear and bacterial genomes;

iv) Infection with *Wolbachia *can sweep the genetic variation on both mitochondrial and nuclear DNAs of the hosts;

v) Infection age correlates with recovery of genetic diversity of host genomes and also with the high prevalence of *Wolbachia *in its populations.

To test these hypotheses we carried out a study on the genetic diversity of the species *Naupactus cervinus*, as part of a broader research project on the evolution of parthenogenesis in South American weevils of the tribe Naupactini. This pest insect, commonly known as the Fuller's rose weevil, is very attractive for studying the evolution of asexuality, because it is worldwide distributed and it is very abundant in nature. Although probably native to Northeastern Argentina, Southern Brazil and Uruguay, in currently occurs in Australia, Azores Islands, Canary Islands, Chile, France, Italy, Japan, Mexico, Morocco, New Zealand, Spain, USA, *etc*. as a consequence of commercial trade of several crops, especially ornamental and fruit plants [29-31].

*Naupactus cervinus *reproduces by parthenogenesis, although some sexual lineages have been recorded from Northeastern Argentina and Southern Brazil in the 1940's (see [32]). Rodriguero *et al*. [19] and Rodriguero [33] reported that all asexual lineages of this species are infected with the *Wolbachia *strain *wNau5*, which belongs to the supergroup B.

The main goal of this contribution is to understand the conquences of *Wolbachia *infection and asexuality on the genomes of *N. cervinus *through a mito-nuclear genetic comparison. To test the hypotheses previously formulated, particularly the occurrence of apomixis, we will quantify the minimum number of recombination events and the linkage disequilibrium between both genomes. Additionally, the estimation of the number of *Wolbachia *strains, their prevalence and the infection age, will bring insights on the influence of this endosymbiont on both host genomes, and will contribute to explain the distribution of their genetic variation.

## Results

### Genetic variation estimates

Three hundred and nine individuals from 38 different locations were screened for genetic variation in a 748 bp fragment of the *COI *gene. All of them were females. Seventeen mitochondrial haplotypes (arbitrarily named A-R [GenBank: GQ406827 - GQ406843]) and 25 segregating sites were identified, of which only three were singletons. Some haplotypes were found in multiple locations (*e.g*. haplotype B, Table 1), while others occurred at a single site (*e.g*. haplotypes A, D, E, G, H, J, L, N, P, Table 1).

**Table 1:** Geographic distribution and genetic diversity of *Naupactus cervinus *samples

Sampling location	Acronym	Lat/Long	N	Infection *Status*	mtDNA haplotypes	nDNA haplotypes	Multilocus genotypes
Alegrete (BR)	Al	29° 46'S, 55° 47'W	5	√	F	VI VIII *	1? *, 1 ? VIII, 3 F VI
Brazo Largo(AR)	BL	33° 54'S, 58° 53'W	11	√	C M	VII *	4 C *, 7 M VII
Bozzano (BR)	Bo	28° 35'S, 53° 59'W	4	√	C Q	II	1 C II, 3 Q II
Buenos Aires (AR)	BA	34° 36' S, 58° 26' W	5	√	B F H	VI	1 B ?, 3 F VI, 1 H VI,
Cardales (AR)	Ca	34° 18'S, 58° 57' W	5	√	B G	VI VII	3 B VII, 2 G VI
Cerro Azul (AR)	CA	27° 38' S, 55° 30' W	10	√	Q	IV	10 Q IV
Chajarí (AR)	Chj	30° 47' S, 57° 59' W	6	√	F	VI VII	5 F VI, 1 F VII
El Palmar (AR)	EP	31° 50' S, 58° 17'W	14	√	F	VI	14 F VI
Easter Island (CH)	IP	27° 08' S, 109° 26' W	7	√	I	---	7 I ?
French Polynesia	PF	23° 08' S, 134° 58' W	1	√	B	VII	1 B VII
Godoy Cruz (AR)	GC	32° 56' S, 68° 50' W	3	√	A	VII	3 A VII
Gualeguaychú (AR)	Gu	33° 01' S, 58° 31' W	18	√	F M	VI VII	13 F VI, 5 M VII
Ijui (BR)	Ij	28° 23' S, 53° 54' W	3	√	C R	*	2 C *, 1 R *
Itaára (BR)	It	29° 36' S, 53° 45' W	4	√	C	VIII *	1 C VIII, 3 C *
Jari (BR)	Ja	29° 17' S, 54° 13' W	3	√	C	*	3 C *
La Falda (AR)	LF	31° 05' S, 64° 29' W	13	√	B	VII	13 B VII
Laranjeiras do Sul (BR)	LS	25° 24' S, 52° 24' W	2	√	R	*	2 R *
Libertad (UR)	Li	34° 37' S, 56° 37' W	2	√	B	VI	2 B VI
Mendoza (AR)	Me	33° 30' S, 69° 00' W	3	√	B	VII	3 B VII
Oberá (AR)	Ob	27° 29' S, 55° 08' W	2	√	Q R	III	1 Q III, 1 R ?
Otamendi Res. (AR)	RO	34° 14' S, 58° 52' W	21	√	N	VII	21 N VII
P. P. Pereyra Iraola (AR)	PI	34° 50' S, 58° 8' W	13	√	B D F	VI VII	1 B VII, 9 D VII, 3 F VI
Pergamino (AR)	Pe	33° 54' S, 60° 35' W	2	√	B	VII	2 B VII
Ponta Grossa (BR)	PG	25° 05' S, 50° 09' W	4	√	C F R	I VI *	1 C *, 2 F VI, 1 R I
Río Cuarto (AR)	RC	33° 08' S, 64° 21' W	5	√	A B	VII	3 A VII, 2 B VII
Salto Grande (AR)	SG	31° 23' S, 58° 01' W	12	√	F	VI	12 F VI
Santa Maria (BR)	SM	29° 40' S, 53° 47' W	10	√	Q	IV	10 Q IV
Santiago de Chile (CH)	SC	33°26' S, 70°29' W	8	√	B	VII	8 B VII
Sao Sepé (BR)	SS	30° 10' S, 53° 34' W	11	√	P	IV	11 P IV
Tahiti	Th	17° 52'S, 149° 56'W	3	√	B	VII	3 B VII
Talavera Island (AR)	IT	34° 10' S, 58° 30' W	19	√	B F K M	VI VII	2 B VII, 6 F VI, 1 K VII, 10 M VII
Tandil(AR)	Ta	37° 19' S, 59° 08' W	18	√	B F L	V VI	5 B V, 10 F VI, 3 L ?
Tenerife (SP)	Te	27° 27' N, 16° 14' W	5	√	B	VII	5 B VII
Toledo (BR)	To	24° 42' S, 53° 44' W	5	√	C	*	5 C *
Tres Lomas (AR)	TL	36° 28' S, 62° 52' W	8	√	B	VII	8 B VII
Valencia (SP)	Val	39° 29' N, 00° 23' W	12	√	B	VII	12 B VII
Vallenar (CH)	Var	28° 57' S, 71° 15' W	11	√	I J	VII	4 I VII, 7 J VII
Yapeyú (AR)	Ya	29° 28' S, 56° 50' W	4	√	C E	*	3 C *, 1 E *
Zárate (AR)	Za	34° 06' S, 59° 01' W	20	√	B K	VII	1 B VII, 19 K VII

Sampling sites of *Naupactus cervinus*. Acronyms, latitude, longitude, sampling size, *Wolbachia *infection *status*, mitochondrial and nuclear haplotypes and multilocus genotypes are specified for each location. The asterisk indicates *ITS1 *"double string" sequences. Whenever one of the haplotypes could not be obtained, it was indicated through a question mark.

**Figure 1:**
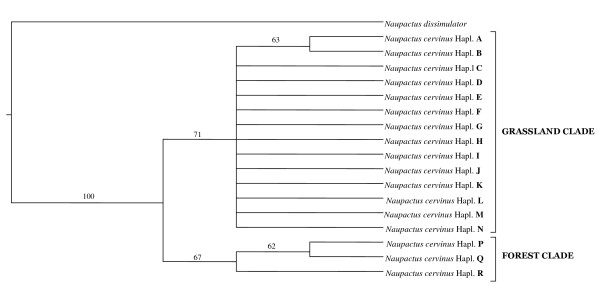
**Mitochondrial diversity**. Strict consensus of 13 most parsimoniuos trees of *COI *haplotypes. Numbers above the branches are 50% or higher bootstrap values.

Alignment of the translated *COI *sequences showed a distribution of the genetic variation similar to that reported by Lunt *et al*. [34]. The absence of *stop *codons and mutations that alter the reading frame excluded *numt *amplifications. The total proportions of nucleotides were 31.8% A, 17.0% C, 15.7% G and 35.6% T, with a strong A + T bias (67.4%), which is higher in the third codon position. Estimates of genetic variation for the total sample were θ_π _= 0.007 ± 0.001, θ*_W _*= 0.007 ± 0.002 and *Hd *= 0.844 ± 0.011. Maximum parsimony search yielded 13 most parsimonious trees 113 steps long. A strict consensus tree is shown, where two main clades have been recovered (Figure 1). One of these clades includes mitochondrial haplotypes from forests ("forest clade"), and the other one mitochondrial haplotypes from open vegetation areas ("grassland clade"), except "F", "G" and "H" that come from a transition zone along the Uruguay River (Figure 1, Table 1). The most parsimonious trees differentiate in minor changes in the relationships among haplotypes within the "grassland clade", yielding a highly unresolved consensus tree with several branches of zero length (*i.e*. a large polytomy).

Sequencing of *ITS1 *in 282 individuals yielded a fragment of *ca*. 1,100 bp, 155 bp of it belonging to the highly conserved *18 S rDNA *region, and the remaining to the *ITS1 *region. Most specimens analyzed were a subsample of those sequenced for *COI*. Twenty three of these individuals showed chromatograms with double peaks ("double string sequences") (Table 1). This finding was indicative of at least two simultaneously amplified sequences from the same individual.

**Figure 2:**
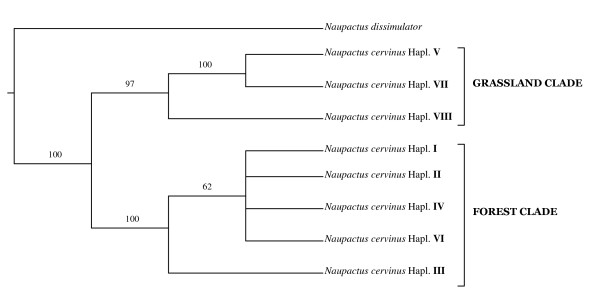
**Nuclear diversity**. Cladogram of *ITS1 *haplotypes. Numbers above the branches are 50% or higher bootstrap values.

Forty-four segregating sites were identified for *ITS1 *(only four singletons) yielding eight different nuclear haplotypes (arbitrarily named I-VIII [GenBank: GQ406818 - GQ406825]). Unlike *COI*, insertion/deletion events were frequent in this dataset. However, based on a gap insertion:substitution cost ratio 10:1, primary homologies could be unambiguously established. Nuclear haplotype distribution depicted a pattern similar to *COI (e.g*. haplotypes VI and VII are widely distributed, and haplotypes I, II and III are restricted to a single site, Table 1). The following values of genetic variability were obtained: θ_π _= 0.026 ± 0.001, θ*_W _*= 0.013 ± 0.002 and *Hd *= 0.574 ± 0.028. The *Hd *value was remarkably lower than that obtained for *COI*, in agreement with the minor number of *ITS1 *haplotypes. Maximum parsimony search yielded one most parsimonious tree 153 steps long (Figure 2). In agreement with the mitochondrial dataset, two divergent clades were recovered: one from open vegetation areas ("grassland clade") and the other from forests ("forest clade"), although the later includes a single haplotype (VI) from the transition zone previously mentioned.

#### Linkage Disequilibrium Analysis

Direct analysis of multilocus genotypes (*COI *and *ITS1*) in 282 individuals shows that in most cases, nuclear and mitochondrial genotypes cosegregate (Table 1). In fact, ten out of 17 mitochondrial haplotypes (A, D, G, H, I, J, K, M, N and P) from 104 individuals are exclusively associated with a single nuclear haplotype. For instance, 20 individuals bearing the mitochondrial haplotype "K" always carry the nuclear haplotype "VII", 11 individuals with "P" always carry "IV", 22 individuals with "M" always carry "VII", and so on (see Table 1). Additionally, the most frequent haplotypes "B" and "F" are linked with nuclear haplotypes "VII" and "VI" respectively, in 118 out of 126 individuals (Table 1).

**Figure 3:**
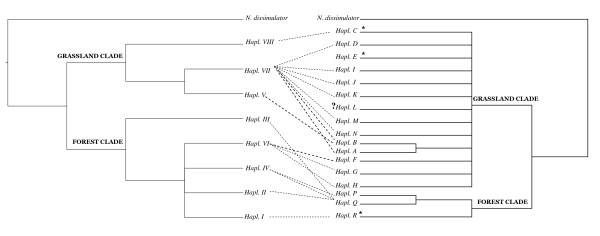
**Phylogenetic congruence**. Congruent phylogenetic relationships between mitochondrial and nuclear haplotypes of *N. cervinus*.

Moreover, the analysis of congruence between *COI *and *ITS1 *gene trees reveals signatures of coevolution between both genomes. For instance, eight mitochondrial haplotypes belonging to the "grassland clade" ("A", "B", "D", "I", "J", "K", "M" and "N") share the grassland nuclear haplotype "VII" (Figure 3). The remaining haplotypes from this same clade "F", "G" and "H, which occur in a transition zone between forests and grasslands (Table 1), are linked to the nuclear haplotype "VI". More interestingly, within the mitochondrial "forest clade" the sister haplotypes "P" and "Q" share the forest nuclear haplotype "IV", and the "R" haplotype bears the nuclear genotype "I", phylogenetically related to "IV" (Figure 3). The inverse association was also observed, since some related nuclear haplotypes are linked to the same mitochondrial haplotype. For example "II", "III" and "IV" are linked with "Q", whereas "V" and "VII" are associated with "B" (Figure 3).

Only four individuals show traces of recombination. They are those carrying the haplotype combinations "B-VI" (N = 2), "C-II" (N = 1) and "F-VII" (N = 1). Moroever, *ITS1 *double strings sequences were found in three mitotypes ("C", "E" and "R"), a fact that could be the consequence of hybridization events.

The hypothesis of mito-nuclear coevolution is also supported by the result of the statistical assessment of the genetic linkage between both genomes. In fact, the 1,403 comparisons of nuclear *vs*. mitochondrial sites yielded 772 significant values of linkage disequilibrium (see Additional File 1, Table S1). After Bonferroni's correction, the number of significant associations decreased to 606. This led to the rejection of the null hypothesis of linkage equilibrium between the nuclear and mitochondrial fragments herein sequenced.

### Analysis of recombination in nuclear sequences

The estimation of the recombination parameter according to R_M _indicates lack of recombination in the nuclear sequence under study (R_M _= 0, *p *= 0). Although *ITS1 *is a small fragment in comparison with the whole nuclear genome, this result may be regarded as an indirect evidence of apomixis. Then, the null hypothesis of apomixis cannot be rejected.

### Selective sweeps

The *MK *test, based on polymorphism and divergence, showed a significant deficit of fixed non-synonymous differences in *COI *(G = 8.199; *p *< 0.01). Then, purifying or negative selection penalizes the non-synonymous substitutions between *N. cervinus *and *N. dissimulator*. The NI and the α parameter support this conclusion (NI = 9.474; α = -8.474). Tajima's *D_T _*and Fu and Li's *F *were nonsignificant (*D_T _*= 0.183, p > 0.05 and *F *= 1.151, p > 0.10), suggesting that genetic variation of *COI *is under selective neutrality or in drift-mutation equilibrium.

The models selected by MrModelTest were GTR + G [35,36] for the mitochondrial dataset, and HKY85 + I [37,38] for the nuclear dataset. Bayesian phylogenetic analyses converged after 100,000 generations, based on the inspection of the burn-in plot of log-likelihood scores, tree lengths, all model parameters, and the analysis of cumulative posterior probabilities and the standard deviation of split frequencies. Therefore, the first 250 samples from each analysis were discarded, resulting in two posterior distributions containing 750 samples each. A plot of the posterior probabilities of all splits from the two separate MCMCMC runs demonstrated a linear relationship, suggesting that these analyses were not restricted to local optima. The *ITS1 *sites were optimized onto this phylogram. The likelihood ratio tests obtained were LRT_1 _= 1.001 (*p *= 0.606) for test 1, and LRT_2 _= 2.001 (*p *= 0.317) for test 2. Therefore, the null hypothesis of no positive selection could not be rejected for the nuclear dataset. In fact, out of the 838 nuclear sites examined, 837 were under negative selection (0.99 >*p *> 0.50) and only one was under positive selection (*p *> 0.99) (data not shown). On the other hand, Tajima's *D_T _*and Fu and Li's *F *were significantly positive for *ITS1 (D_T _*= 2.979, p < 0.01 and *F *= 2.193, p < 0.02), not accounting for a selective sweep.

### Demographic analysis

ModelTest selected the GTR model for *ITS1 *[35]. The values of the parameters and confidence intervals (α = 0.05) were: *g *= -1.748 [(-4.950)-(-0.289)] and θ = 0.004 [0.002-0.008]. The sign of *g *indicates that *N. cervinus *most likely passed through a bottleneck (*i.e*. a decline in population size). In turn, the equation of *g *implies that θ was higher in the past.

### Prevalence and diversity of *Wolbachia *in *Naupactus cervinus*

A total of 247 individuals of *N. cervinus *from different locations were screened by PCR assay using *Wolbachia 16 S rDNA *gene-specific primers. All of them were positive for *Wolbachia *infection, as it was demonstrated by the amplification of a product of approximately 800 bp. This fragment size was similar to that of the positive control. No PCR band was obtained from the negative control.

**Figure 4:**
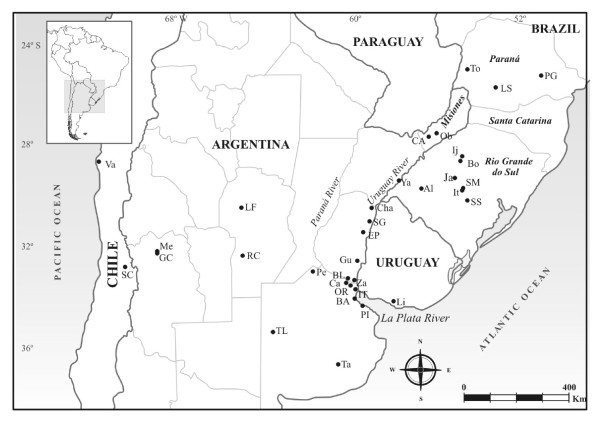
**Geographic distribution of *Naupactus cervinus***. Sampling locations of *Naupactus cervinus*. Countries included in this study are indicated in capital letter.

To investigate multiple infections within *N. cervinus*, we focused on 16 sampling sites, including weevils from both the "forest" and the "grassland" clades (Brazo Largo, Cerro Azul, Chajarí, El Palmar, Isla Talavera, Oberá, Pergamino, Río Cuarto, Salto Grande, Tandil and Yapeyú from Argentina, and Alegrete, Laranjeiras do Sul, Santa Maria, São Sepé and Toledo from Brazil, Figure 4). Sixteen randomly chosen gene fragments *wsp, coxA *and *fbpA *were amplified (one from every sampling site) and sequenced. All the individuals assayed yielded identical gene sequences (*wsp: ca*. 580 bp in length [GenBank: GQ402145]; *coxA: ca*. 400 bp in length [GenBank: GU079631.2]; *fbpA ca*. 430 bp in length [GenBank: GU079632.1]). Based on these results, we concluded that only one *Wolbachia *strain infects this weevil species. Therefore, although multiple infections could have occurred, only one would have succeded.

### Divergence time estimation

The age of the demographic event (*i.e*. moderate bottleneck) was calculated through a molecular clock, using both the nuclear and the mitochondrial datasets.

Table 2 shows a matrix of uncorrected genetic distances among all the nuclear haplotypes (I-VIII). The relative rate tests indicate that all these nDNA lineages may have split at a constant substitution rate (p > 0.10 for all comparisons). The average pairwise divergence among them was D = 0.03198, so the divergence time for this dataset would be *ca*. 1.9 Myrs.

**Table 2:** Nuclear divergence

	I	II	III	IV	V	VI	VII	VII
I	---							
II	**0.05170**	---						
III	**0.05296**	**0.00883**	---					
IV	**0.04918**	**0.00504**	**0.00378**	---				
V	**0.05044**	**0.00631**	**0.00504**	**0.00126**	---			
VI	**0.05044**	**0.00631**	**0.00504**	**0.00126**	**0.00252**	---		
VII	**0.03657**	**0.05296**	**0.05422**	**0.05044**	**0.05170**	**0.05170**	---	
VIII	**0.03657**	**0.05296**	**0.05422**	**0.05044**	**0.05170**	**0.05170**	**0.00000**	---

Uncorrected genetic distances among nuclear haplotypes of *N. cervinus*. The values with constant substitution rate, as verified by the relative-rate test, are in bold (*p *> 0.10).

Table 3 shows a matrix of uncorrected genetic distances among all the mitochondrial haplotypes (A-R). As in the former case, the relative rate test indicate that all these mtDNA lineages may have splitted at a constant substitution rate (p > 0.10 for all comparisons). Divergence time among all mitochondrial haplotypes is more recent than that obtained for the nuclear dataset. Based on an average distance D = 0.00962, it was 400,000 years. The difference between both markers will be discussed later.

**Table 3:** Mitochondrial divergence

	A	B	C	D	E	F	G	H	I	J	K	L	M	N	P	Q	R
A	---																
B	**0.00000**	---															
C	**0.00174**	**0.00174**	---														
D	**0.00348**	**0.00348**	**0.00174**	---													
E	**0.00348**	**0.00348**	**0.00174**	**0.00348**	---												
F	**0.00348**	**0.00348**	**0.00174**	**0.00348**	**0.00348**	---											
G	**0.00522**	**0.00522**	**0.00348**	**0.00522**	**0.00522**	**0.00174**	---										
H	**0.00696**	**0.00522**	**0.00522**	**0.00696**	**0.00696**	**0.00348**	**0.00174**	---									
I	**0.00522**	**0.00522**	**0.00348**	**0.00522**	**0.00522**	**0.00522**	**0.00348**	**0.00522**	---								
J	**0.00522**	**0.00522**	**0.00348**	**0.00522**	**0.00522**	**0.00522**	**0.00348**	**0.00522**	**0.00348**	---							
K	**0.00348**	**0.00348**	**0.00174**	**0.00348**	**0.00348**	**0.00348**	**0.00174**	**0.00348**	**0.00174**	**0.00174**	---						
L	**0.00522**	**0.00522**	**0.00348**	**0.00522**	**0.00522**	**0.00522**	**0.00348**	**0.00522**	**0.00348**	**0.00348**	**0.00174**	---					
M	**0.00348**	**0.00348**	**0.00174**	**0.00348**	**0.00348**	**0.00348**	**0.00174**	**0.00348**	**0.00174**	**0.00174**	**0.00000**	**0.00174**	---				
N	**0.00522**	**0.00522**	**0.00348**	**0.00522**	**0.00522**	**0.00522**	**0.00348**	**0.00522**	**0.00348**	**0.00348**	**0.00174**	**0.00348**	**0.00174**	---			
P	**0.02261**	**0.02261**	**0.02087**	**0.02261**	**0.02261**	**0.02261**	**0.02087**	**0.02261**	**0.02087**	**0.02087**	**0.01913**	**0.02087**	**0.01913**	**0.02087**	---		
Q	**0.02435**	**0.02435**	**0.02261**	**0.02435**	**0.02435**	**0.02435**	**0.02261**	**0.02435**	**0.02261**	**0.02261**	**0.02087**	**0.02261**	**0.02087**	**0.02261**	**0.00174**	---	
R	**0.02435**	**0.02435**	**0.02261**	**0.02435**	**0.02435**	**0.02435**	**0.02261**	**0.02435**	**0.02261**	**0.02261**	**0.02087**	**0.02261**	**0.02087**	**0.02261**	**0.00870**	**0.01043**	---

Uncorrected genetic distances among mitochondrial haplotypes of *N. cervinus*. The values with constant substitution rate, as verified by the relative-rate test, are in bold (*p *> 0.10).

The *wsp *is the most rapid evolving gene in *Wolbachia *[39], but strickingly, it remains invariant in all the individuals assayed. Assuming 0.002 changes per site per Myrs, the *wsp *sequence should have accumulated 0.0008-0.0036 changes per site in 0.4-1.9 Myrs. In a sequence *ca*. 580 bp in lenght, we should have found 0.46-2 changes. Therefore, invariancy is not an unexpected result.

## Discussion

The estimates of mtDNA genetic variation of *N. cervinus *are similar to those reported for other species of Curculionidae having similar life history traits (*e.g*. [40-42]). There is a remarkable bias in the nucleotide proportions of mtDNA toward A/T, mainly in the third positions, which is very common in insects [43,44], including weevils (*e.g*. [41,45-49]).

The comparison of the haplotype diversity between the two markers herein investigated suggests that the substitution rate of the nuclear genome would be slower than that of the mitochondrial genome, as it has been reported for several animal species (see [50] for a review), including *N. cervinus *[51].

Apomixis implies clonal transmission of all genomes and lack of recombination. As a consequence, this kind of asexual reproduction will induce cosegregation of mitochondrial and nuclear genotypes and the consequent linkage disequilibrium among all genetic markers [52,53]. The cosegregation of nuclear and mitochondrial haplotypes seen in most individuals herein analyzed provides evidence of the clonal transmission of both genomes in *N. cervinus*. Furthermore, the congruence between *ITS1 *and *COI *phylogenetic trees indicates that coevolution between these genomes have occurred, since many phylogenetically related mitotypes share the same nuclear genotype or viceversa. This phylogenetic congruence suggests that an ancestral mitochondrial haplotype and all its derived lineages evolved along with the same nuclear haplotype across evolutionary times, possibly as a consequence of the higher substitution rate for the mitochondrial gene. Therefore, the hypothesis of ancient linkage disequilibrium and long history of mito-nuclear genomic association is strongly supported.

Only a few number of individuals (1.4%) showed signals of recombination. Besides, traces of hybridization were inferred from individuals carrying at least two nuclear haplotypes or "double strings" *ITS1 *sequences (8.2%) (a more detailed study of this exciting finding is currently being undertaken). These individuals could be either remnants of historical gene flow, or the consequence of occasional crosses between parthenogenetic females of *N. cervinus *and males of the same or other related species (as it was reported by Saura *et al*. [17] and Stenberg *et al*. [54] for other weevils with similar reproductive behavior). However, these sporadic sexual events would not invalidate the hypothesis of apomixis for this weevil, since there is clonal transmission of nuclear and mitochondrial genomes, lack of recombination (R_M _= 0) and non-random association for several mitochondrial and nuclear sites.

The cytoplasmic location of the *Wolbachia *endosymbiont and the mitochondrial organelle will naturally induce linkage disequilibrium among all their genetic markers. Furthermore, the non-random association among mitochondrial and nuclear markers would indicate that the nuclear genome is also linked with the other two. Then, the selective sweep on mtDNA induced by *Wolbachia *should have affected also the nuclear genome.

If the initial *Wolbachia *infection have sweeped the mitochondrial diversity, a significant and negative Tajima's *D_T_*, and a significant and negative NI should be expected. However, the first statistics suggests selective neutrality and the second, negative selection. The neutral result from Tajima's *D_T _*test could be explained by the antiquity of the infection. Most probably, the time elapsed since this event had been long enough to recover the genetic variation to levels prior to the initial selective sweep, increasing *Hd *and θ*_W _*values. A similar result was reported by Shoemaker *et al*. [55], Keller *et al*. [56] and Marshall [57], who proposed ancient infections for *Solenopsis invicta *(Hymenoptera, Formicidae), *Chelymorpha alternans *(Coleoptera, Chrysomelidae) and *Allonemobius fasciatus-socius *species complex (Orthoptera, Gryllidae). On the other hand, the apparent contrasting result of the *MK *test (*i.e*. negative selection) might be interpreted under the Nearly Neutral Theory [27,58-62]. In fact, the value for this statistic would indicate an excess of deleterous mutations, which according to the mentioned theory, would be fixed by chance only if the effective population size is small. In other words, the selective pressure coefficients that operate on the *COI *gene would be so small (*i.e*. nearly neutral), that elimination cannot occur.

Low nucleotide diversity in the mitochondrial genome is expected in recently infected species [25,26]. However, a comparison with the related sexual species *Naupactus xanthographus*, which is unifected [19], would indicate that both species exhibit comparable levels of mitochondrial genetic diversity [63]. This result is interpreted as another evidence of the ancient *Wolbachia *infection in *N. cervinus*.

Regarding the nuclear gene, even though *ITS1 *is a non-functional region, it does not evolve freely in insects [64]. In sexual species, *ca*. 40% of nucleotide sites are implied in complex secondary structures, and hence under natural selection [65]. Among the 838 sites analyzed herein, 837 showed evidence of negative selection, which is overwhelming in comparison with the result of Schlötterer *et al*. [65]. This bias in the sites under negative selection could be explained by the genetic linkage among the three genomes, in agreement with Schön *et al*. [52]. Then, the negative selection operating on bacterial, mitochondrial or nuclear genes could also drive other linked nuclear sites to a similar fate.

The positive Tajima's *D_T _*obtained for the nuclear gene may suggest a balancing selection, a spatial population structure of the genetic variation or a moderate bottleneck (after Depaulis *et al*. [66]). The fact that directional negative selection was detected for *ITS1 *rules out the first possibility and points to a demographic phenomenon as the most likely explanation for the *D_T _*result. Indeed, the LAMARC analysis supported this conclusion, suggesting that *N. cervinus *could have suffered a bottleneck, probably as a consequence of the *Wolbachia *invasion [67]. The moderate perturbation in the population size of the initial uninfected population could have allowed some lineages to survive this invasion.

Why this demographic phenomenon was not detected for the mitochondrial gene? The mitochondrial genome usually undergoes faster evolution than the nuclear genome (*e.g*. [68,69]). Thus, the signatures of the bottleneck could have been erased in *N. cervinus *mtDNA. Then, the significant Tajima's *D_T _*suggests that the bottleneck was probably ancient, since the traces of this demographic event are still recovered from the nuclear dataset, but not from the mitochondrial one.

The fact that all the individuals tested were infected with the same *Wolbachia *strain (identified by three genes of rapid evolution) favors the hypothesis that the infection of *N. cervinus *occurred once, and that all extant haplotypes of this weevil species are descended from a single ancestral infected female. However, the positive Tajima's *D_T _*for nDNA suggests a different scenario, according to which at least two genetic lineages had been infected with the same strain.

The divergence time estimated from mitochondrial and nuclear datasets allowed us to infer that the bottleneck in *N. cervinus *population occurred between 400,000-1,900,000 years ago, *i.e*. during the Plio-Pleistocene, a geological period of enormous cyclical changes in the South American forests and grasslands [70,71]. The older age obtained for nDNA can be attributed to those lineages that survived the demographic event, which could produce an overestimation of the calculated age. On the other side, the younger age inferred for the mitochondrial dataset can be attributed to the faster accumulation of genetic variation, which yielded younger lineages that decreases the mean distance, leading to an underestimation of the age. Therefore, nDNA and mtDNA estimates can be considered as the upper and the lower age limits of the demographic crash.

The amount of evolutionary change accumulated in mtDNA is very high in contrast to the lack of change in *wsp*, which is the most rapidly evolving gene known in *Wolbachia *[39]. However, according to the age estimated for the infection in *N. cervinus, wsp *might have accumulated *ca*. 0.46-2 substitutions in 0.4-1.9 Myrs. So, lack of variation is not an unexpected result. A similar *wsp *invariant pattern was found in other organisms like sandflies, leaf beetles and fruitflies (*e.g*. [25,56,72]. An alternative explanation could be that substitution rates for mitochondrial and nuclear genomes are different from those assumed herein, due to asexual reproduction. In fact, acceleration [73] and deceleration [52] of substitution rates have been reported for species with clonal reproduction. The first alternative implies a more recent infection, in agreement with the lack of variation of *wsp Wolbachia *gene. However, a decelerated substitution rate would indicate an age of infection much older than that herein estimated.

Although our present data do not allow an accurate inference of the infection age, the high prevalence of *Wolbachia *in the whole species distribution contradict the hypothesis of a recent invasion. In fact, although prevalence of *Wolbachia *is usually related to different phenotypes [74], it can also be associated to the infection age [23]. As *Wolbachia *was detected in 100% of the individuals of *N. cervinus *assayed, its high prevalence is compatible with the hypothesis of an ancient infection.

Our results suggest that *Wolbachia *"lived together" with its host *N. cervinus *at least for a third of its lifespan [see [33]]. If the origin of the apomictic lineages were related to this infection, could *Wolbachia *be responsible for the extinction of all the bisexual populations of *N. cervinus*? Given that some males were collected in 1945 and 1947 in the forests of Northern Argentina and Southern Brazil [32], it is unlikely that this bacterium caused their extinction. Rather, the intense deforestation of the Paranaense forest that occurred during the last 40 years [75] is the most important factor to explain the extinction of the bisexual populations of this weevil.

## Conclusion

We provide first genetic evidence of apomixis for the weevil *Naupactus cervinus*. This kind of parthenogenesis, in addition to *Wolbachia *infection, induces linkage disequilibrium among three genomes: the nuclear and mitochondrial weevil genomes and the bacterial genome. Hence, the mito-nuclear genetic variation of the host would have been shaped by apomictic reproduction, the moderate bottleneck probably caused by the initial *Wolbachia *infection, the long time passed since then, and the high prevalence of the unique *Wolbachia *strain infecting this weevil.

Even if *Wolbachia *accounts for the origin of parthenogenesis in *N. cervinus*, this infection would have not caused the extinction of the bisexual population. The current absence of males and the lack of sexual reproduction during the last 50 years would be a consequence of the destruction of the native forest where these populations occurred.

## Methods

### Sampling and specimens examined

Adults of *N. cervinus *(Entiminae: Naupactini) were collected during the summers of 2004-2007, on wild and cultivated plants from several geographic locations of Argentina, Southern Brazil and Uruguay. Samples from the countries where the species has been introduced were also included (see Figure 4 and Table 1) (N = 309). Specimens were collected using a beating sheet (55 cm × 55 cm) and stored at -80°C or in 100% ethanol at 4°C for molecular analyses.

### PCR assay and sequencing

Total genomic DNA was extracted following the protocol of Reiss *et al*. [76]. The negative controls were samples lacking DNA template.

A segment of *ca*. 700 bp of the Cytochrome Oxidase I (*COI*) mitochondrial gene of *N. cervinus *was amplified using the specific primers S1718 (5'-GGA GGA TTT GGA AAT TGA TTA GTT CC-3') and A2442 (5'-GCT AAT CAT CTA AAA ATT TTA ATT CCT GTT GG-3') [77] and a nuclear region of *ca*. 1100 bp using the primers rDNA2 (5'-TTG ATT ACG TCC CTG CCC TTT-3') [78] and rDNA 1.58 S (5'-ACG AGC CGA GTG ATC CAC CG-3') [79], which are suitable for amplifying the region 3' of the *18 S rDNA *gene, plus the complete *ITS1 *region (*Internal Transcribed Spacer *1) and the 5' region of the *5.8 S rDNA *gene.

Search of multiple *Wolbachia *lineages within *N. cervinus *was accomplished through amplification and sequencing of the *wsp *gene fragment using the primers brought by Braig *et al*. [80]: *wsp *81F (5'-TGG TCC AAT AAG TGA TGA AGA AAC-3') and *wsp *691R (5'-AAA AAT TAA ACG CTA CTC CA -3'), and the most variables MLST genes for the B supergroup *coxA *and *fbpA*, using the primers designed by Baldo *et al*. [81]: coxA F1 (5'-TTG GRG CRA TYA ACT TTA TAG-3'), coxA R1(5'-CTA AAG ACT TTK ACR CCA GT-3'), fbpA F1 (5'-GCT GCT CCR CTT GGY WTG AT-3') and fbpA R1 (5'-CCR CCA GAR AAA AYY ACT ATT C-3').

Prevalence of *Wolbachia *infection was studied through amplification of *16 S rDNA *gene in ten weevils (whenever it was possible) from every sampling site, using the primers designed by O'Neill *et al*. [82]: forward (5'-TTG TAG CCT GCT ATG GTA TAA CT-3') and *reverse *(5'-GAA TAG GTA TGA TTT TCA TGT-3').

Total genomic DNA from *Drosophila melanogaster *naturally infected with *Wolbachia *was used as a positive control. Negative controls consisted of samples lacking DNA template from insects and *D. melanogaster *treated with tetracyline. *D. melanogaster *DNA was kindly provided by Dr. Scott O'Neill (Queensland University, Australia). All experiments were repeated at least twice.

Amplification was carried out in a 50 μl volume reaction with 50-100 ng of DNA used as template, 0.5 μM of each primer, 0.1 mM of each dNTP, 3.0 mM MgCl_2_, 0.05 units of Taq polymerase and reaction buffer 1× (Invitrogen). The reactions were performed in a GeneAmp^® ^PCR System 2700 thermal cycler (Applied Biosystems) under the conditions described by Scataglini *et al*. [48] for *COI *and Szalanski and Owens [83] for *ITS1 *fragments.

Double-stranded PCR products were separated by electrophoresis on a 1% agarose gel with TAE buffer containing 0.5 mg⁄ml of ethidium bromide. The bands were excised from the gel and the DNA was purified with a QIAquick Gel Extraction Kit (Qiagen Inc.). DNA was sequenced using a 3130-XL Automatic Sequencer (Applied Biosystems).

### Sequence analysis

Standard chromatographic curves of forward and reverse sequences were edited using the Bioedit program [84]. Sequences were translated with the program MEGA v. 4.0.1 [85] to check for the presence of stop codons or frame shifts which might indicate the amplification of pseudogenes [86-89]. Aminoacid sequences were inferred according to the invertebrate mitochondrial code [34]. Alignment was done using CLUSTAL W [90] and adjusted by eye.

### Quantifying genetic variability

Nucleotide diversity for each gene region was estimated using Watterson's (θ*_W_*) [91] and Tajima's (θ_π_) [92] estimators. Haplotype diversity (*Hd*) was also estimated according to Nei [93]. All the calculations were performed with DnaSP v. 5.10.00 [94].

Phylogenetic analyses of mitochondrial and nuclear haplotypes were performed by maximum parsimony using haplotypes as "terminal taxa" with the program NONA v. 2.0 [95], executed through the interface WinClada v. 1.00.08 [96]. The implicit enumeration search option was used to get the most parsimonious trees (number of taxa < 25). All characters were regarded as unordered and unweighted. For the nuclear dataset, gaps were treated as fifth state. Clade stability was assessed by 10,000 parsimony bootstrap replications [97]. *Naupactus dissimulator *was sequenced to use it as outgroup [GenBank: GQ406844 for *COI *and GQ406826 for *ITS1*].

### Detecting Linkage disequilibrium

Significant associations between mtDNA and nDNA sites were tested using linkage disequilibrium analysis based on the *D *parameter [98]. This parameter was obtained with DnaSP v. 5.10.00 [94]. Bonferroni's correction for multiple comparisons [99,100] was used for avoiding spurious rejections of the null hypothesis (*i.e*. linkage equilibrium).

### Detecting Recombination

The population recombination parameter was estimated with the method of Hudson and Kaplan [101] based on the minimum number of recombination events in a sample (R_M_) using DnaSP v. 5.10.00 [94]. The significance of the test was calculated by performing 10,000 coalescent simulations based on a Monte Carlo process with no recombination [102].

### Investigating selective sweeps

Tajima's *D_T _*[103] and Fu and Li's *F *[104] test statistics were applied to evaluate whether the frequency spectrum of segregating sites departs from neutral expectations [105] for both datasets. In addition, the *MK *test [106] was applied to evaluate a possible correlation between polymorphism and divergence for the synonymous to non-synonymous variation ratio predicted by the Neutral Theory [105] only for the mtDNA dataset (coding sequences). The neutrality index (NI) [107] and the α parameter [108] were used as indicators of the degree and direction of departures from neutrality; NI = 1 indicates strict neutrality and NI < 1 suggests an excess of non-synonymous fixation (*i.e*. adaptive evolution). The α parameter reflects the proportion of fixed mutations by positive selection. Unlike *D_T_, MK *is highly recommended when demographic factors can obscure the results of neutrality tests [27,109,110]. These estimations were performed with DnaSP v. 5.10.00 [94], using the same outgroup species as mentioned above.

For the non-conding nDNA dataset, the approach developed by Wong and Nielsen [111] was used. It is a maximum likelihood method for inferring positive selection in non-coding regions, by comparing non-coding divergence *vs*. synonymous divergence. This approach assumes that synonymous mutations are neutral and occur at a constant rate within a coding fragment. The ratio of the estimated nucleotide substitution rate in the non-coding region to the estimated synonymous substitution rate from the coding region (ξ), provides a numerical approach to assess whether a non-coding region has evolved by positive selection. The unit of evolution is one nucleotide, and ξ is the nucleotide substitution rate in the non-coding region normalized by the synonymous nucleotide substitution rate in the coding region. Therefore, when a site is subject to neutral selection, ξ = 1. Similarly, ξ > 1 indicates positive selection, while ξ < 1 suggests the occurrence of negative selection.

Three models are implemented in the EvoNC software [111]. The neutral, the two-category, and the three-category models were used in two different tests to determine whether a non-coding region is under positive selection. The Test 1 compares the neutral *vs*. the three category model, and the Test 2, less conservative, compares the neutral *vs*. the two-category model [111]. The two mentioned tests were applied to identify non-coding sites under positive selection, with the coding sites of the mtDNA being considered as linked to the *ITS1 *sites. Therefore, mitochondrial sites were used to estimate the synonymous evolution rate because nuclear and mitochondrial genomes were assumed to be linked to each other and also to *Wolbachia *genome.

For obtaining ξ, the nucleotide positions must be optimized on a phylogenetic tree which includes the sequences under analysis (*i.e*. the mitochondrial and nuclear haplotypes concatenated). A matrix of concatenated sequences was built whenever the *COI *and *ITS1 *fragments came from the same individual. This matrix had 21 terminals (including *N. dissimulator *as outgroup) of 1,521 bp in length (683 bp *COI *+ 838 bp *ITS1*).

The phylogenetic tree was estimated through Bayesian inference. MrModeltest software v. 2.2 [112] was used to infer the most appropriate model of molecular evolution for each dataset based on the Akaike Information Criterion (AIC) [113,114], as suggested by Posada and Buckley [115].

Bayesian phylogenetic analysis was performed using the "Metropolis-coupled Markov chain Monte Carlo" (MCMCMC) algorithm implemented in MrBayes ver. 3.1.2 [116,117]. A partitioned algorithm was used to account for heterogeneity between the two datasets. Program defaults were used for estimation of priors. Two independent analyses were run using a random starting tree with three heated chains and one cold chain over 400,000 generations, with sampling every 100 generations.

The tree space was explored using four chains: one cold chain and three incrementally heated ones, heat being set as 1/(1 + (*i *-1) T), where *i *is the chain number (*i.e*. 1-4) and *T *is used as default.

Stationarity of the Bayesian analysis was evaluated with the methodologies and statistics implemented in Tracer [118] and AWTY [119,120] and with the standard deviation of the split frequencies. All posterior samples of a run prior to this point were discarded as burn-in. Remaining trees were used to construct a 50% majority-rule consensus tree with mean branch length estimates. The frequency of all observed bipartitions was used to assess the level of support for each node [116,117].

### Analyzing demographic structure

Tajima's test *D_T _*[103] results can reveal either selective or demographic events. To disentangle the demographic factors for the interpretation of this test, it was necessary to demonstrate that a population bottleneck had occurred. For this purpose we use the program LAMARC [121] to estimate the relative effective population size parameter (θ) and the exponential growth rate (*g*) by maximum likelihood, as well as the calculation of 95% confidence intervals. Since the maximum likelihood estimation procedure is computationally intensive, 60 individuals were selected randomly from each dataset because results do not change significantly at this sample size [122]. Positive values of *g *indicate population growth, while negative values indicate shrinkage. The estimation of the parameters was done using the MCMCMC sampling algorithm for *ITS1* implemented in LAMARC v. 2.0 [121]. The analysis consisted of two simultaneous searches with heating temperature adjusted automatically with 10 initial chains of 5,000 steps sampled every 20 steps, followed by two final chains of 50,000 steps sampled every 20 steps. Final most likely estimates (MLEs) were calculated using parameter estimates from two replicated analyses.

The evolution model was selected using the program ModelTest [123] as previously described. According to Kuhner *et al*. [122], the inclusion of unlinked *loci *reduces the bias in estimating *g *with a small amount of individuals. As the reproductive mode of *N. cervinus *makes this recommendation meaningless, the sign of *g *was analyzed without taking into account its absolute value.

### Estimating divergence times

Molecular clock estimates were calculated through the mean divergence time among all nuclear and mitochondrial haplotypes found for *N. cervinus*. Relative-rate tests were performed to test the equality of evolutionary rates between lineages [124] using MEGA v. 4.0.1 [85]. The divergence times were calculated from the average of the uncorrected pairwise genetic distances among haplotypes with MEGA v. 4.0.1, using the equation *T = D*/2*k *(where *T *is the mean divergence time, *D *is the mean number of pairwise differences per site, and *k *is the estimated rate of nucleotide substitution). We decided not to apply substitution models to correct the pairwise distances, because we considered that the levels of divergence among haplotypes within the same species is low enough to deserve a correction. In order to estimate these divergence times, we used a nucleotide substitution rate of 0.85% per site per Myrs for *ITS1 *[68] and 1.2% per site per Myrs for *COI *[125].

Additionally, we calculated the expected variation for *Wolbachia wsp *gene assuming a nucleotide substitution rate of 0.2% [126].

## Authors' contributions

MSR carried out the molecular work, analized the data and wrote a first draft of the manuscript. All authors contributed to obtain samples of specimens and to the final analyses and writing of the manuscript.

## Supplementary Material

Additional file 1 **Table S1: Linkage disequilibrium test**. Values of the *D *parameter. The significant disequilibrium linkage after Bonferroni's correction is indicated by the letter B. * *p *< 0. 005; ** *p *< 0. 010; *** *p *< 0. 001 Click here for file
