# Supplementary material for: Mito-nuclear genetic comparison in a Wolbachia infected weevil: insights on reproductive mode, infection age and evolutionary forces shaping genetic variation
Source: BMC Evol Biol. 2010 Nov 4;10:340. doi: 10.1186/1471-2148-10-340 (PMC2992525; doi:10.1186/1471-2148-10-340)
Supplement: Additional file 1 — Table S1: Linkage disequilibrium test. Values of the D parameter. The significant disequilibrium linkage after Bonferroni's correction is indicated by the letter B. * p < 0. 005; ** p < 0. 010; *** p < 0. 001 [file 1471-2148-10-340-S1.PDF]

ADDITIONAL FILE

Table S1: Linkage disequilibrium test

mtDNA sites	nDNA sites	*D*	χ^2	mtDNA sites	nDNA sites	*D*	χ^2
59	745	0. 006	2. 815	59	1373	0. 000	0. 011
59	749	0. 006	2. 815	59	1378	-0. 003	0. 688
59	757	0. 000	0. 011	59	1383	0. 005	93. 997***B
59	769	0. 005	93. 997***B	59	1386	0. 006	2. 815
59	776	0. 006	2. 815	59	1398	0. 006	2. 815
59	788	0. 006	2. 815	59	1433	0. 000	0. 011
59	794	0. 001	0. 064	59	1472	0. 006	2. 815
59	798	0. 006	2. 754	59	1490	0. 006	2. 815
59	804	0. 006	2. 815	59	1495	0. 006	2. 815
59	814	-0. 003	0. 688	59	1502	0. 006	2. 754
59	829	0. 000	0. 011	69	745	-0. 004	1. 452
59	838	0. 006	2. 754	69	749	-0. 004	1. 452
59	856	0. 006	2. 815	69	757	0. 000	0. 011
59	870	0. 006	2. 815	69	769	0. 000	0. 011
59	872	0. 000	0. 011	69	776	-0. 004	1. 452
59	882	0. 006	2. 815	69	788	-0. 004	1. 452
59	902	0. 006	2. 754	69	794	-0. 004	1. 420
59	907	0. 006	2. 754	69	798	-0. 004	1. 484
59	923	0. 006	2. 815	69	804	-0. 004	1. 452
59	934	0. 000	0. 011	69	814	-0. 003	0. 688
59	947	0. 006	2. 754	69	829	0. 000	0. 011
59	948	-0. 003	0. 688	69	838	-0. 004	1. 484
59	951	0. 000	0. 011	69	856	-0. 004	1. 452
59	952	0. 000	0. 011	69	870	-0. 004	1. 452
59	954	0. 006	2. 815	69	872	0. 000	0. 011
59	969	0. 000	0. 011	69	882	-0. 004	1. 452
59	973	0. 000	0. 011	69	902	-0. 004	1. 484
59	975	0. 006	2. 815	69	907	-0. 004	1. 484
59	977	0. 000	0. 011	69	923	-0. 004	1. 452
59	978	0. 000	0. 011	69	934	0. 000	0. 011
59	982	0. 006	2. 754	69	947	-0. 004	1. 484
59	983	0. 006	2. 815	69	948	-0. 003	0. 688
59	990	0. 000	0. 011	69	951	0. 000	0. 011
59	1074	0. 000	0. 011	69	952	0. 000	0. 011
59	1078	0. 000	0. 044	69	954	-0. 004	1. 452
59	1114	0. 006	2. 815	69	969	0. 000	0. 011
59	1117	0. 006	2. 815	69	973	0. 000	0. 011
59	1167	0. 006	2. 754	69	975	-0. 004	1. 452
59	1181	0. 006	2. 815	69	977	0. 000	0. 011
59	1200	0. 006	2. 815	69	978	0. 000	0. 011
59	1228	0. 006	2. 754	69	982	-0. 004	1. 484
59	1243	0. 006	2. 815	69	983	-0. 004	1. 452
59	1247	0. 006	2. 754	69	990	0. 000	0. 011
59	1273	0. 006	2. 754	69	1074	0. 000	0. 011
59	1277	0. 005	93. 997***B	69	1078	0. 000	0. 044
59	1295	0. 006	2. 754	69	1114	-0. 004	1. 452
59	1334	0. 006	2. 754	69	1117	-0. 004	1. 452
59	1337	0. 006	2. 754	69	1167	-0. 004	1. 484
59	1340	0. 006	2. 815	69	1181	-0. 004	1. 452

59	1346	0. 006	2. 754	69	1200	-0. 004	1. 452
59	1370	0. 005	93. 997***B	69	1228	-0. 004	1. 484
69	1243	-0. 004	1. 452	72	1117	0. 068	34. 668***B
69	1247	-0. 004	1. 484	72	1167	0. 067	33. 924***B
69	1273	-0. 004	1. 484	72	1181	0. 068	34. 668***B
69	1277	0. 000	0. 011	72	1200	0. 068	34. 668***B
69	1337	-0. 004	1. 484	72	1228	0. 067	33. 924***B
69	1340	-0. 004	1. 452	72	1243	0. 068	34. 668***B
69	1346	-0. 004	1. 484	72	1247	0. 067	33. 924***B
69	1370	0. 000	0. 011	72	1273	0. 067	33. 924***B
69	1373	0. 000	0. 011	72	1277	-0. 001	0. 132
69	1378	-0. 003	0. 688	72	1295	0. 067	33. 924***B
69	1383	0. 000	0. 011	72	1334	0. 067	33. 924***B
69	1386	-0. 004	1. 452	72	1337	0. 067	33. 924***B
69	1398	-0. 004	1. 452	72	1340	0. 068	34. 668***B
69	1433	0. 000	0. 011	72	1346	0. 067	33. 924***B
69	1472	-0. 004	1. 452	72	1370	-0. 001	0. 132
69	1490	-0. 004	1. 452	72	1373	-0. 001	0. 132
69	1495	-0. 004	1. 452	72	1378	-0. 030	8. 476**
69	1502	-0. 004	1. 484	72	1383	-0. 001	0. 132
72	745	0. 068	34. 668***B	72	1386	0. 068	34. 668***B
72	749	0. 068	34. 668***B	72	1398	0. 068	34. 668***B
72	757	-0. 001	0. 132	72	1433	-0. 001	0. 132
72	769	-0. 001	0. 132	72	1472	0. 068	34. 668***B
72	776	0. 068	34. 668***B	72	1490	0. 068	34. 668***B
72	788	0. 068	34. 668***B	72	1495	0. 068	34. 668***B
72	794	0. 068	35. 432***B	72	1502	0. 067	33. 924***B
72	798	0. 067	33. 924***B	120	745	0. 092	49. 654***B
72	804	0. 068	34. 668***B	120	749	0. 092	49. 654***B
72	814	-0. 030	8. 476**	120	757	-0. 001	0. 190
72	829	-0. 001	0. 132	120	769	0. 004	5. 328*
72	838	0. 067	33. 924***B	120	776	0. 092	49. 654***B
72	856	0. 068	34. 668***B	120	788	0. 092	49. 654***B
72	870	0. 068	34. 668***B	120	794	0. 088	45. 150***B
72	872	-0. 001	0. 132	120	798	0. 092	48. 587***B
72	882	0. 068	34. 668***B	120	804	0. 092	49. 654***B
72	902	0. 067	33. 924***B	120	814	-0. 040	12. 140***
72	907	0. 067	33. 924***B	120	829	-0. 001	0. 190
72	923	0. 068	34. 668***B	120	838	0. 092	48. 587***B
72	934	-0. 001	0. 132	120	856	0. 092	49. 654***B
72	947	0. 067	33. 924***B	120	870	0. 092	49. 654***B
72	948	-0. 030	8. 476**	120	872	-0. 001	0. 190
72	951	-0. 001	0. 132	120	882	0. 092	49. 654***B
72	952	-0. 001	0. 132	120	902	0. 092	48. 587***B
72	954	0. 068	34. 668***B	120	907	0. 092	48. 587***B
72	969	-0. 001	0. 132	120	923	0. 092	49. 654***B
72	973	-0. 001	0. 132	120	934	-0. 001	0. 190
72	975	0. 068	34. 668***B	120	947	0. 092	48. 587***B
72	977	-0. 001	0. 132	120	948	-0. 040	12. 140***
72	978	-0. 001	0. 132	120	951	-0. 001	0. 190
72	982	0. 067	33. 924***B	120	952	-0. 001	0. 190
72	983	0. 068	34. 668***B	120	954	0. 092	49. 654***B
72	990	-0. 001	0. 132	120	969	-0. 001	0. 190
72	1074	-0. 001	0. 132	120	973	-0. 001	0. 190
72	1078	0. 013	15. 950***	120	975	0. 092	49. 654***B
72	1114	0. 068	34. 668***B	120	977	-0. 001	0. 190

69	1295	-0. 004	1. 484
69	1334	-0. 004	1. 484
120	983	0. 092	49. 654***B
120	990	-0. 001	0. 190
120	1074	-0. 001	0. 190
120	1078	0. 013	10. 699**
120	1114	0. 092	49. 654***B
120	1117	0. 092	49. 654***B
120	1167	0. 092	48. 587***B
120	1181	0. 092	49. 654***B
120	1200	0. 092	49. 654***B
120	1228	0. 092	48. 587***B
120	1243	0. 092	49. 654***B
120	1247	0. 092	48. 587***B
120	1273	0. 092	48. 587***B
120	1277	0. 004	5. 328*
120	1295	0. 092	48. 587***B
120	1334	0. 092	48. 587***B
120	1337	0. 092	48. 587***B
120	1340	0. 092	49. 654***B
120	1346	0. 092	48. 587***B
120	1370	0. 004	5. 328*
120	1373	-0. 001	0. 190
120	1378	-0. 040	12. 140***
120	1383	0. 004	5. 328*
120	1386	0. 092	49. 654***B
120	1398	0. 092	49. 654***B
120	1433	-0. 001	0. 190
120	1472	0. 092	49. 654***B
120	1490	0. 092	49. 654***B
120	1495	0. 092	49. 654***B
120	1502	0. 092	48. 587***B
162	745	-0. 005	0. 065
162	749	-0. 005	0. 065
162	757	-0. 002	0. 754
162	769	0. 003	1. 34
162	776	-0. 005	0. 065
162	788	-0. 005	0. 065
162	794	-0. 008	0. 182
162	798	-0. 007	0. 146
162	804	-0. 005	0. 065
162	814	-0. 093	35. 207***B
162	829	-0. 002	0. 754
162	838	-0. 007	0. 146
162	856	-0. 005	0. 065
162	870	-0. 005	0. 065
162	872	-0. 002	0. 754
162	882	-0. 005	0. 065
162	902	-0. 007	0. 146
162	907	-0. 007	0. 146
162	923	-0. 005	0. 065
162	934	-0. 002	0. 754
162	947	-0. 007	0. 146
162	948	-0. 093	35. 207***B
162	951	-0. 002	0. 754
162	952	-0. 002	0. 754
120	978	-0. 001	0. 190
120	982	0. 092	48. 587***B
162	973	-0. 002	0. 754
162	975	-0. 005	0. 065
162	977	-0. 002	0. 754
162	978	-0. 002	0. 754
162	982	-0. 007	0. 146
162	983	-0. 005	0. 065
162	990	-0. 002	0. 754
162	1074	-0. 002	0. 754
162	1078	0. 007	1. 724
162	1114	-0. 005	0. 065
162	1117	-0. 005	0. 065
162	1167	-0. 007	0. 146
162	1181	-0. 005	0. 065
162	1200	-0. 005	0. 065
162	1228	-0. 007	0. 146
162	1243	-0. 005	0. 065
162	1247	-0. 007	0. 146
162	1273	-0. 007	0. 146
162	1277	0. 003	1. 340
162	1295	-0. 007	0. 146
162	1334	-0. 007	0. 146
162	1337	-0. 007	0. 146
162	1340	-0. 005	0. 065
162	1346	-0. 007	0. 146
162	1370	0. 003	1. 340
162	1373	-0. 002	0. 754
162	1378	-0. 093	35. 207***B
162	1383	0. 003	1. 340
162	1386	-0. 005	0. 065
162	1398	-0. 005	0. 065
162	1433	-0. 002	0. 754
162	1472	-0. 005	0. 065
162	1490	-0. 005	0. 065
162	1495	-0. 005	0. 065
162	1502	-0. 007	0. 146
202	745	-0. 024	8. 388**
202	749	-0. 024	8. 388**
202	757	0. 000	0. 062
202	769	0. 000	0. 062
202	776	-0. 024	8. 388**
202	788	-0. 024	8. 388**
202	794	-0. 024	8. 207**
202	798	-0. 025	8. 572**
202	804	-0. 024	8. 388**
202	814	-0. 015	3. 976*
202	829	0. 000	0. 062
202	838	-0. 025	8. 572**
202	856	-0. 024	8. 388**
202	870	-0. 024	8. 388**
202	872	0. 000	0. 062
202	882	-0. 024	8. 388**
202	902	-0. 025	8. 572**
202	907	-0. 025	8. 572**
202	923	-0. 024	8. 388**

162	954	-0. 005	0. 065
162	969	-0. 002	0. 754
202	948	-0. 015	3. 976*
202	951	0. 000	0. 062
202	952	0. 000	0. 062
202	954	-0. 024	8. 388**
202	969	0. 000	0. 062
202	973	0. 000	0. 062
202	975	-0. 024	8. 388**
202	977	0. 000	0. 062
202	978	0. 000	0. 062
202	982	-0. 025	8. 572**
202	983	-0. 024	8. 388**
202	990	0. 000	0. 062
202	1074	0. 000	0. 062
202	1078	-0. 001	0. 253
202	1114	-0. 024	8. 388**
202	1117	-0. 024	8. 388**
202	1167	-0. 025	8. 572**
202	1181	-0. 024	8. 388**
202	1200	-0. 024	8. 388**
202	1228	-0. 025	8. 572**
202	1243	-0. 024	8. 388**
202	1247	-0. 025	8. 572**
202	1273	-0. 025	8. 572**
202	1277	0. 000	0. 062
202	1295	-0. 025	8. 572**
202	1334	-0. 025	8. 572**
202	1337	-0. 025	8. 572**
202	1340	-0. 024	8. 388**
202	1346	-0. 025	8. 572**
202	1370	0. 000	0. 062
202	1373	0. 000	0. 062
202	1378	-0. 015	3. 976*
202	1383	0. 000	0. 062
202	1386	-0. 024	8. 388**
202	1398	-0. 024	8. 388**
202	1433	0. 000	0. 062
202	1472	-0. 024	8. 388**
202	1490	-0. 024	8. 388**
202	1495	-0. 024	8. 388**
202	1502	-0. 025	8. 572**
204	745	0. 092	49. 654***B
204	749	0. 092	49. 654***B
204	757	-0. 001	0. 190
204	769	0. 004	5. 328*
204	776	0. 092	49. 654***B
204	788	0. 092	49. 654***B
204	794	0. 088	45. 150***B
204	798	0. 092	48. 587***B
204	804	0. 092	49. 654***B
204	814	-0. 04	12. 140***
204	829	-0. 001	0. 190
204	838	0. 092	48. 587***B
204	856	0. 092	49. 654***B
204	870	0. 092	49. 654***B
202	934	0. 000	0. 062
202	947	-0. 025	8. 572**
204	902	0. 092	48. 587***B
204	907	0. 092	48. 587***B
204	923	0. 092	49. 654***B
204	934	-0. 001	0. 190
204	947	0. 092	48. 587***B
204	948	-0. 040	12. 140***
204	951	-0. 001	0. 190
204	952	-0. 001	0. 190
204	954	0. 092	49. 654***B
204	969	-0. 001	0. 190
204	973	-0. 001	0. 190
204	975	0. 092	49. 654***B
204	977	-0. 001	0. 190
204	978	-0. 001	0. 190
204	982	0. 092	48. 587***B
204	983	0. 092	49. 654***B
204	990	-0. 001	0. 190
204	1074	-0. 001	0. 190
204	1078	0. 013	10. 699**
204	1114	0. 092	49. 654***B
204	1117	0. 092	49. 654***B
204	1167	0. 092	48. 587***B
204	1181	0. 092	49. 654***B
204	1200	0. 092	49. 654***B
204	1228	0. 092	48. 587***B
204	1243	0. 092	49. 654***B
204	1247	0. 092	48. 587***B
204	1273	0. 092	48. 587***B
204	1277	0. 004	5. 328*
204	1295	0. 092	48. 587***B
204	1334	0. 092	48. 587***B
204	1337	0. 092	48. 587***B
204	1340	0. 092	49. 654***B
204	1346	0. 092	48. 587***B
204	1370	0. 004	5. 328*
204	1373	-0. 001	0. 190
204	1378	-0. 040	12. 140***
204	1383	0. 004	5. 328*
204	1386	0. 092	49. 654***B
204	1398	0. 092	49. 654***B
204	1433	-0. 001	0. 190
204	1472	0. 092	49. 654***B
204	1490	0. 092	49. 654***B
204	1495	0. 092	49. 654***B
204	1502	0. 092	48. 587***B
256	745	0. 006	2. 815
256	749	0. 006	2. 815
256	757	0. 000	0. 011
256	769	0. 005	93. 997***B
256	776	0. 006	2. 815
256	788	0. 006	2. 815
256	794	0. 001	0. 064
256	798	0. 006	2. 754
256	804	0. 006	2. 815

204	872	-0. 001	0. 190
204	882	0. 092	49. 654***B
256	838	0. 006	2. 754
256	856	0. 006	2. 815
256	870	0. 006	2. 815
256	872	0. 000	0. 011
256	882	0. 006	2. 815
256	902	0. 006	2. 754
256	907	0. 006	2. 754
256	923	0. 006	2. 815
256	934	0. 000	0. 011
256	947	0. 006	2. 754
256	948	-0. 003	0. 688
256	951	0. 000	0. 011
256	952	0. 000	0. 011
256	954	0. 006	2. 815
256	969	0. 000	0. 011
256	973	0. 000	0. 011
256	975	0. 006	2. 815
256	977	0. 000	0. 011
256	978	0. 000	0. 011
256	982	0. 006	2. 754
256	983	0. 006	2. 815
256	990	0. 000	0. 011
256	1074	0. 000	0. 011
256	1078	0. 000	0. 044
256	1114	0. 006	2. 815
256	1117	0. 006	2. 815
256	1167	0. 006	2. 754
256	1181	0. 006	2. 815
256	1200	0. 006	2. 815
256	1228	0. 006	2. 754
256	1243	0. 006	2. 815
256	1247	0. 006	2. 754
256	1273	0. 006	2. 754
256	1277	0. 005	93. 997***B
256	1295	0. 006	2. 754
256	1334	0. 006	2. 754
256	1337	0. 006	2. 754
256	1340	0. 006	2. 815
256	1346	0. 006	2. 754
256	1370	0. 005	93. 997***B
256	1373	0. 000	0. 011
256	1378	-0. 003	0. 688
256	1383	0. 005	93. 997***B
256	1386	0. 006	2. 815
256	1398	0. 006	2. 815
256	1433	0. 000	0. 011
256	1472	0. 006	2. 815
256	1490	0. 006	2. 815
256	1495	0. 006	2. 815
256	1502	0. 006	2. 754
264	745	0. 092	49. 654***B
264	749	0. 092	49. 654***B
264	757	-0. 001	0. 190
264	769	0. 004	5. 328*

256	814	-0. 003	0. 688
256	829	0. 000	0. 011
264	794	0. 088	45. 150***B
264	798	0. 092	48. 587***B
264	804	0. 092	49. 654***B
264	814	-0. 04	12. 140***
264	829	-0. 001	0. 190
264	838	0. 092	48. 587***B
264	856	0. 092	49. 654***B
264	870	0. 092	49. 654***B
264	872	-0. 001	0. 190
264	882	0. 092	49. 654***B
264	902	0. 092	48. 587***B
264	907	0. 092	48. 587***B
264	923	0. 092	49. 654***B
264	934	-0. 001	0. 190
264	947	0. 092	48. 587***B
264	948	-0. 04	12. 140***
264	951	-0. 001	0. 190
264	952	-0. 001	0. 190
264	954	0. 092	49. 654***B
264	969	-0. 001	0. 190
264	973	-0. 001	0. 190
264	975	0. 092	49. 654***B
264	977	-0. 001	0. 190
264	978	-0. 001	0. 190
264	982	0. 092	48. 587***B
264	983	0. 092	49. 654***B
264	990	-0. 001	0. 190
264	1074	-0. 001	0. 190
264	1078	0. 013	10. 699**
264	1114	0. 092	49. 654***B
264	1117	0. 092	49. 654***B
264	1167	0. 092	48. 587***B
264	1181	0. 092	49. 654***B
264	1200	0. 092	49. 654***B
264	1228	0. 092	48. 587***B
264	1243	0. 092	49. 654***B
264	1247	0. 092	48. 587***B
264	1273	0. 092	48. 587***B
264	1277	0. 004	5. 328*
264	1295	0. 092	48. 587***B
264	1334	0. 092	48. 587***B
264	1337	0. 092	48. 587***B
264	1340	0. 092	49. 654***B
264	1346	0. 092	48. 587***B
264	1370	0. 004	5. 328*
264	1373	-0. 001	0. 190
264	1378	-0. 040	12. 140***
264	1383	0. 004	5. 328*
264	1386	0. 092	49. 654***B
264	1398	0. 092	49. 654***B
264	1433	-0. 001	0. 190
264	1472	0. 092	49. 654***B
264	1490	0. 092	49. 654***B
264	1495	0. 092	49. 654***B

264	776	0. 092	49. 654***B
264	788	0. 092	49. 654***B
303	749	-0. 112	48. 230***B
303	757	-0. 001	0. 392
303	769	-0. 001	0. 392
303	776	-0. 112	48. 230***B
303	788	-0. 112	48. 230***B
303	794	-0. 11	47. 133***B
303	798	-0. 113	49. 346***B
303	804	-0. 112	48. 230***B
303	814	-0. 066	21. 487***B
303	829	-0. 001	0. 392
303	838	-0. 113	49. 346***B
303	856	-0. 112	48. 230***B
303	870	-0. 112	48. 230***B
303	872	-0. 001	0. 392
303	882	-0. 112	48. 230***B
303	902	-0. 113	49. 346***B
303	907	-0. 113	49. 346***B
303	923	-0. 112	48. 230***B
303	934	-0. 001	0. 392
303	947	-0. 113	49. 346***B
303	948	-0. 066	21. 487***B
303	951	-0. 001	0. 392
303	952	-0. 001	0. 392
303	954	-0. 112	48. 230***B
303	969	-0. 001	0. 392
303	973	-0. 001	0. 392
303	975	-0. 112	48. 230***B
303	977	-0. 001	0. 392
303	978	-0. 001	0. 392
303	982	-0. 113	49. 346***B
303	983	-0. 112	48. 230***B
303	990	-0. 001	0. 392
303	1074	-0. 001	0. 392
303	1078	-0. 006	1. 593
303	1114	-0. 112	48. 230***B
303	1117	-0. 112	48. 230***B
303	1167	-0. 113	49. 346***B
303	1181	-0. 112	48. 230***B
303	1200	-0. 112	48. 230***B
303	1228	-0. 113	49. 346***B
303	1243	-0. 112	48. 230***B
303	1247	-0. 113	49. 346***B
303	1273	-0. 113	49. 346***B
303	1277	-0. 001	0. 392
303	1295	-0. 113	49. 346***B
303	1334	-0. 113	49. 346***B
303	1337	-0. 113	49. 346***B
303	1340	-0. 112	48. 230***B
303	1346	-0. 113	49. 346***B
303	1370	-0. 001	0. 392
303	1373	-0. 001	0. 392
303	1378	-0. 066	21. 487***B
303	1383	-0. 001	0. 392
303	1386	-0. 112	48. 230***B
264	1502	0. 092	48. 587***B
303	745	-0. 112	48. 230***B
303	1472	-0. 112	48. 230***B
303	1490	-0. 112	48. 230***B
303	1495	-0. 112	48. 230***B
303	1502	-0. 113	49. 346***B
312	745	0. 092	49. 654***B
312	749	0. 092	49. 654***B
312	757	-0. 001	0. 190
312	769	0. 004	5. 328*
312	776	0. 092	49. 654***B
312	788	0. 092	49. 654***B
312	794	0. 088	45. 150***B
312	798	0. 092	48. 587***B
312	804	0. 092	49. 654***B
312	814	-0. 040	12. 140***
312	829	-0. 001	0. 190
312	838	0. 092	48. 587***B
312	856	0. 092	49. 654***B
312	870	0. 092	49. 654***B
312	872	-0. 001	0. 190
312	882	0. 092	49. 654***B
312	902	0. 092	48. 587***B
312	907	0. 092	48. 587***B
312	923	0. 092	49. 654***B
312	934	-0. 001	0. 190
312	947	0. 092	48. 587***B
312	948	-0. 040	12. 140***
312	951	-0. 001	0. 19
312	952	-0. 001	0. 190
312	954	0. 092	49. 654***B
312	969	-0. 001	0. 190
312	973	-0. 001	0. 190
312	975	0. 092	49. 654***B
312	977	-0. 001	0. 190
312	978	-0. 001	0. 190
312	982	0. 092	48. 587***B
312	983	0. 092	49. 654***B
312	990	-0. 001	0. 190
312	1074	-0. 001	0. 190
312	1078	0. 013	10. 699**
312	1114	0. 092	49. 654***B
312	1117	0. 092	49. 654***B
312	1167	0. 092	48. 587***B
312	1181	0. 092	49. 654***B
312	1200	0. 092	49. 654***B
312	1228	0. 092	48. 587***B
312	1243	0. 092	49. 654***B
312	1247	0. 092	48. 587***B
312	1273	0. 092	48. 587***B
312	1277	0. 004	5. 328*
312	1295	0. 092	48. 587***B
312	1334	0. 092	48. 587***B
312	1337	0. 092	48. 587***B
312	1340	0. 092	49. 654***B
312	1346	0. 092	48. 587***B

303	1398	-0. 112	48. 230***B
303	1433	-0. 001	0. 392
312	1378	-0. 040	12. 140***
312	1383	0. 004	5. 328*
312	1386	0. 092	49. 654***B
312	1398	0. 092	49. 654***B
312	1433	-0. 001	0. 190
312	1472	0. 092	49. 654***B
312	1490	0. 092	49. 654***B
312	1495	0. 092	49. 654***B
312	1502	0. 092	48. 587***B
313	745	0. 086	45. 768***B
313	749	0. 086	45. 768***B
313	757	-0. 001	0. 175
313	769	-0. 001	0. 175
313	776	0. 086	45. 768***B
313	788	0. 086	45. 768***B
313	794	0. 087	46. 776***B
313	798	0. 085	44. 785***B
313	804	0. 086	45. 768***B
313	814	-0. 038	11. 190***
313	829	-0. 001	0. 175
313	838	0. 085	44. 785***B
313	856	0. 086	45. 768***B
313	870	0. 086	45. 768***B
313	872	-0. 001	0. 175
313	882	0. 086	45. 768***B
313	902	0. 085	44. 785***B
313	907	0. 085	44. 785***B
313	923	0. 086	45. 768***B
313	934	-0. 001	0. 175
313	947	0. 085	44. 785***B
313	948	-0. 038	11. 190***
313	951	-0. 001	0. 175
313	952	-0. 001	0. 175
313	954	0. 086	45. 768***B
313	969	-0. 001	0. 175
313	973	-0. 001	0. 175
313	975	0. 086	45. 768***B
313	977	-0. 001	0. 175
313	978	-0. 001	0. 175
313	982	0. 085	44. 785***B
313	983	0. 086	45. 768***B
313	990	-0. 001	0. 175
313	1074	-0. 001	0. 175
313	1078	0. 013	11. 729***
313	1114	0. 086	45. 768***B
313	1117	0. 086	45. 768***B
313	1167	0. 085	44. 785***B
313	1181	0. 086	45. 768***B
313	1200	0. 086	45. 768***B
313	1228	0. 085	44. 785***B
313	1243	0. 086	45. 768***B
313	1247	0. 085	44. 785***B
313	1273	0. 085	44. 785***B
313	1277	-0. 001	0. 175

312	1370	0. 004	5. 328*
312	1373	-0. 001	0. 190
313	1337	0. 085	44. 785***B
313	1340	0. 086	45. 768***B
313	1346	0. 085	44. 785***B
313	1370	-0. 001	0. 175
313	1373	-0. 001	0. 175
313	1378	-0. 038	11. 190***
313	1383	-0. 001	0. 175
313	1386	0. 086	45. 768***B
313	1398	0. 086	45. 768***B
313	1433	-0. 001	0. 175
313	1472	0. 086	45. 768***B
313	1490	0. 086	45. 768***B
313	1495	0. 086	45. 768***B
313	1502	0. 085	44. 785***B
351	745	0. 143	83. 289***B
351	749	0. 143	83. 289***B
351	757	-0. 001	0. 342
351	769	-0. 001	0. 342
351	776	0. 143	83. 289***B
351	788	0. 143	83. 289***B
351	794	0. 144	85. 181***B
351	798	0. 141	81. 445***B
351	804	0. 143	83. 289***B
351	814	0. 184	178. 592***B
351	829	-0. 001	0. 342
351	838	0. 141	81. 445***B
351	856	0. 143	83. 289***B
351	870	0. 143	83. 289***B
351	872	-0. 001	0. 342
351	882	0. 143	83. 289***B
351	902	0. 141	81. 445***B
351	907	0. 141	81. 445***B
351	923	0. 143	83. 289***B
351	934	-0. 001	0. 342
351	947	0. 141	81. 445***B
351	948	0. 184	178. 592***B
351	951	-0. 001	0. 342
351	952	-0. 001	0. 342
351	954	0. 143	83. 289***B
351	969	-0. 001	0. 342
351	973	-0. 001	0. 342
351	975	0. 143	83. 289***B
351	977	-0. 001	0. 342
351	978	-0. 001	0. 342
351	982	0. 141	81. 445***B
351	983	0. 143	83. 289***B
351	990	-0. 001	0. 342
351	1074	-0. 001	0. 342
351	1078	-0. 005	1. 391
351	1114	0. 143	83. 289***B
351	1117	0. 143	83. 289***B
351	1167	0. 141	81. 445***B
351	1181	0. 143	83. 289***B
351	1200	0. 143	83. 289***B

313	1295	0. 085	44. 785***B
313	1334	0. 085	44. 785***B
351	1247	0. 141	81. 445***B
351	1273	0. 141	81. 445***B
351	1277	-0. 001	0. 342
351	1295	0. 141	81. 445***B
351	1334	0. 141	81. 445***B
351	1337	0. 141	81. 445***B
351	1340	0. 143	83. 289***B
351	1346	0. 141	81. 445***B
351	1370	-0. 001	0. 342
351	1373	-0. 001	0. 342
351	1378	0. 184	178. 592***B
351	1383	-0. 001	0. 342
351	1386	0. 143	83. 289***B
351	1398	0. 143	83. 289***B
351	1433	-0. 001	0. 342
351	1472	0. 143	83. 289***B
351	1490	0. 143	83. 289***B
351	1495	0. 143	83. 289***B
351	1502	0. 141	81. 445***B
357	745	0. 092	49. 654***B
357	749	0. 092	49. 654***B
357	757	-0. 001	0. 190
357	769	0. 004	5. 328*
357	776	0. 092	49. 654***B
357	788	0. 092	49. 654***B
357	794	0. 088	45. 150***B
357	798	0. 092	48. 587***B
357	804	0. 092	49. 654***B
357	814	-0. 040	12. 140***
357	829	-0. 001	0. 190
357	838	0. 092	48. 587***B
357	856	0. 092	49. 654***B
357	870	0. 092	49. 654***B
357	872	-0. 001	0. 190
357	882	0. 092	49. 654***B
357	902	0. 092	48. 587***B
357	907	0. 092	48. 587***B
357	923	0. 092	49. 654***B
357	934	-0. 001	0. 190
357	947	0. 092	48. 587***B
357	948	-0. 040	12. 140***
357	951	-0. 001	0. 190
357	952	-0. 001	0. 190
357	954	0. 092	49. 654***B
357	969	-0. 001	0. 190
357	973	-0. 001	0. 190
357	975	0. 092	49. 654***B
357	977	-0. 001	0. 190
357	978	-0. 001	0. 190
357	982	0. 092	48. 587***B
357	983	0. 092	49. 654***B
357	990	-0. 001	0. 190
357	1074	-0. 001	0. 190
357	1078	0. 013	10. 699**
351	1228	0. 141	81. 445***B
351	1243	0. 143	83. 289***B
357	1167	0. 092	48. 587***B
357	1181	0. 092	49. 654***B
357	1200	0. 092	49. 654***B
357	1228	0. 092	48. 587***B
357	1243	0. 092	49. 654***B
357	1247	0. 092	48. 587***B
357	1273	0. 092	48. 587***B
357	1277	0. 004	5. 328*
357	1295	0. 092	48. 587***B
357	1334	0. 092	48. 587***B
357	1337	0. 092	48. 587***B
357	1340	0. 092	49. 654***B
357	1346	0. 092	48. 587***B
357	1370	0. 004	5. 328*
357	1373	-0. 001	0. 190
357	1378	-0. 040	12. 140***
357	1383	0. 004	5. 328*
357	1386	0. 092	49. 654***B
357	1398	0. 092	49. 654***B
357	1433	-0. 001	0. 190
357	1472	0. 092	49. 654***B
357	1490	0. 092	49. 654***B
357	1495	0. 092	49. 654***B
357	1502	0. 092	48. 587***B
402	745	0. 092	49. 654***B
402	749	0. 092	49. 654***B
402	757	-0. 001	0. 190
402	769	0. 004	5. 328*
402	776	0. 092	49. 654***B
402	788	0. 092	49. 654***B
402	794	0. 088	45. 150***B
402	798	0. 092	48. 587***B
402	804	0. 092	49. 654***B
402	814	-0. 040	12. 140***
402	829	-0. 001	0. 190
402	838	0. 092	48. 587***B
402	856	0. 092	49. 654***B
402	870	0. 092	49. 654***B
402	872	-0. 001	0. 190
402	882	0. 092	49. 654***B
402	902	0. 092	48. 587***B
402	907	0. 092	48. 587***B
402	923	0. 092	49. 654***B
402	934	-0. 001	0. 190
402	947	0. 092	48. 587***B
402	948	-0. 040	12. 140***
402	951	-0. 001	0. 190
402	952	-0. 001	0. 190
402	954	0. 092	49. 654***B
402	969	-0. 001	0. 190
402	973	-0. 001	0. 190
402	975	0. 092	49. 654***B
402	977	-0. 001	0. 190
402	978	-0. 001	0. 190

357	1114	0. 092	49. 654***B
357	1117	0. 092	49. 654***B
402	990	-0. 001	0. 190
402	1074	-0. 001	0. 190
402	1078	0. 013	10. 699**
402	1114	0. 092	49. 654***B
402	1117	0. 092	49. 654***B
402	1167	0. 092	48. 587***B
402	1181	0. 092	49. 654***B
402	1200	0. 092	49. 654***B
402	1228	0. 092	48. 587***B
402	1243	0. 092	49. 654***B
402	1247	0. 092	48. 587***B
402	1273	0. 092	48. 587***B
402	1277	0. 004	5. 328*
402	1295	0. 092	48. 587***B
402	1334	0. 092	48. 587***B
402	1337	0. 092	48. 587***B
402	1340	0. 092	49. 654***B
402	1346	0. 092	48. 587***B
402	1370	0. 004	5. 328*
402	1373	-0. 001	0. 190
402	1378	-0. 040	12. 140***
402	1383	0. 004	5. 328*
402	1386	0. 092	49. 654***B
402	1398	0. 092	49. 654***B
402	1433	-0. 001	0. 190
402	1472	0. 092	49. 654***B
402	1490	0. 092	49. 654***B
402	1495	0. 092	49. 654***B
402	1502	0. 092	48. 587***B
408	745	0. 092	49. 654***B
408	749	0. 092	49. 654***B
408	757	-0. 001	0. 190
408	769	0. 004	5. 328*
408	776	0. 092	49. 654***B
408	788	0. 092	49. 654***B
408	794	0. 088	45. 150***B
408	798	0. 092	48. 587***B
408	804	0. 092	49. 654***B
408	814	-0. 040	12. 140***
408	829	-0. 001	0. 190
408	838	0. 092	48. 587***B
408	856	0. 092	49. 654***B
408	870	0. 092	49. 654***B
408	872	-0. 001	0. 190
408	882	0. 092	49. 654***B
408	902	0. 092	48. 587***B
408	907	0. 092	48. 587***B
408	923	0. 092	49. 654***B
408	934	-0. 001	0. 190
408	947	0. 092	48. 587***B
408	948	-0. 040	12. 140***
408	951	-0. 001	0. 190
408	952	-0. 001	0. 190
408	954	0. 092	49. 654***B
402	982	0. 092	48. 587***B
402	983	0. 092	49. 654***B
408	1200	0. 092	49. 654***B
408	1228	0. 092	48. 587***B
408	1243	0. 092	49. 654***B
408	1247	0. 092	48. 587***B
408	1273	0. 092	48. 587***B
408	1277	0. 004	5. 328*
408	1295	0. 092	48. 587***B
408	1334	0. 092	48. 587***B
408	1337	0. 092	48. 587***B
408	1340	0. 092	49. 654***B
408	1346	0. 092	48. 587***B
408	1370	0. 004	5. 328*
408	1373	-0. 001	0. 190
408	1378	-0. 040	12. 140***
408	1383	0. 004	5. 328*
408	1386	0. 092	49. 654***B
408	1398	0. 092	49. 654***B
408	1433	-0. 001	0. 190
408	1472	0. 092	49. 654***B
408	1490	0. 092	49. 654***B
408	1495	0. 092	49. 654***B
408	1502	0. 092	48. 587***B
408	975	0. 092	49. 654***B
408	977	-0. 001	0. 190
408	978	-0. 001	0. 190
408	982	0. 092	48. 587***B
408	983	0. 092	49. 654***B
408	990	-0. 001	0. 190
408	1074	-0. 001	0. 190
408	1078	0. 013	10. 699**
408	1114	0. 092	49. 654***B
408	1117	0. 092	49. 654***B
408	1167	0. 092	48. 587***B
408	1181	0. 092	49. 654***B
414	745	0. 092	49. 654***B
414	749	0. 092	49. 654***B
414	757	-0. 001	0. 190
414	769	0. 004	5. 328*
414	776	0. 092	49. 654***B
414	788	0. 092	49. 654***B
414	794	0. 088	45. 150***B
414	798	0. 092	48. 587***B
414	804	0. 092	49. 654***B
414	814	-0. 040	12. 140***
414	829	-0. 001	0. 190
414	838	0. 092	48. 587***B
414	856	0. 092	49. 654***B
414	870	0. 092	49. 654***B
414	872	-0. 001	0. 190
414	882	0. 092	49. 654***B
414	902	0. 092	48. 587***B
414	907	0. 092	48. 587***B
414	923	0. 092	49. 654***B
414	934	-0. 001	0. 19

408	969	-0. 001	0. 190
408	973	-0. 001	0. 190
414	951	-0. 001	0. 190
414	952	-0. 001	0. 190
414	954	0. 092	49. 654***B
414	969	-0. 001	0. 190
414	973	-0. 001	0. 190
414	975	0. 092	49. 654***B
414	977	-0. 001	0. 190
414	978	-0. 001	0. 190
414	982	0. 092	48. 587***B
414	983	0. 092	49. 654***B
414	990	-0. 001	0. 190
414	1074	-0. 001	0. 190
414	1078	0. 013	10. 699**
414	1114	0. 092	49. 654***B
414	1117	0. 092	49. 654***B
414	1167	0. 092	48. 587***B
414	1181	0. 092	49. 654***B
414	1200	0. 092	49. 654***B
414	1228	0. 092	48. 587***B
414	1243	0. 092	49. 654***B
414	1247	0. 092	48. 587***B
414	1273	0. 092	48. 587***B
414	1277	0. 004	5. 328*
414	1295	0. 092	48. 587***B
414	1334	0. 092	48. 587***B
414	1337	0. 092	48. 587***B
414	1340	0. 092	49. 654***B
414	1346	0. 092	48. 587***B
414	1370	0. 004	5. 328*
414	1373	-0. 001	0. 190
414	1378	-0. 040	12. 140***
414	1383	0. 004	5. 328*
414	1386	0. 092	49. 654***B
414	1398	0. 092	49. 654***B
414	1433	-0. 001	0. 190
414	1472	0. 092	49. 654***B
414	1490	0. 092	49. 654***B
414	1495	0. 092	49. 654***B
414	1502	0. 092	48. 587***B
417	745	0. 006	2. 815
417	749	0. 006	2. 815
417	757	0. 000	0. 011
417	769	0. 005	93. 997***B
417	776	0. 006	2. 815
417	788	0. 006	2. 815
417	794	0. 001	0. 064
417	798	0. 006	2. 754
417	804	0. 006	2. 815
417	814	-0. 003	0. 688
417	829	0. 000	0. 011
417	838	0. 006	2. 754
417	856	0. 006	2. 815
417	870	0. 006	2. 815
417	872	0. 000	0. 011

414	947	0. 092	48. 587***B
414	948	-0. 040	12. 140***
417	907	0. 006	2. 754
417	923	0. 006	2. 815
417	934	0. 000	0. 011
417	947	0. 006	2. 754
417	948	-0. 003	0. 688
417	951	0. 000	0. 011
417	952	0. 000	0. 011
417	954	0. 006	2. 815
417	969	0. 000	0. 011
417	973	0. 000	0. 011
417	975	0. 006	2. 815
417	977	0. 000	0. 011
417	978	0. 000	0. 011
417	982	0. 006	2. 754
417	983	0. 006	2. 815
417	990	0. 000	0. 011
417	1074	0. 000	0. 011
417	1078	0. 000	0. 044
417	1114	0. 006	2. 815
417	1117	0. 006	2. 815
417	1167	0. 006	2. 754
417	1181	0. 006	2. 815
417	1200	0. 006	2. 815
417	1228	0. 006	2. 754
417	1243	0. 006	2. 815
417	1247	0. 006	2. 754
417	1273	0. 006	2. 754
417	1277	0. 005	93. 997***B
417	1295	0. 006	2. 754
417	1334	0. 006	2. 754
417	1337	0. 006	2. 754
417	1340	0. 006	2. 815
417	1346	0. 006	2. 754
417	1370	0. 005	93. 997***B
417	1373	0. 000	0. 011
417	1378	-0. 003	0. 688
417	1383	0. 005	93. 997***B
417	1386	0. 006	2. 815
417	1398	0. 006	2. 815
417	1433	0. 000	0. 011
417	1472	0. 006	2. 815
417	1490	0. 006	2. 815
417	1495	0. 006	2. 815
417	1502	0. 006	2. 754
420	745	0. 086	45. 768***B
420	749	0. 086	45. 768***B
420	757	-0. 001	0. 175
420	769	-0. 001	0. 175
420	776	0. 086	45. 768***B
420	788	0. 086	45. 768***B
420	794	0. 087	46. 776***B
420	798	0. 085	44. 785***B
420	804	0. 086	45. 768***B
420	814	-0. 038	11. 190***

417	882	0. 006	2. 815	420	829	-0. 001	0. 175
417	902	0. 006	2. 754	420	838	0. 085	44. 785***B
420	856	0. 086	45. 768***B	513	798	-0. 018	6. 131*
420	870	0. 086	45. 768***B	513	804	-0. 018	5. 999*
420	872	-0. 001	0. 175	513	814	-0. 011	2. 844
420	882	0. 086	45. 768***B	513	829	0. 000	0. 044
420	902	0. 085	44. 785***B	513	838	-0. 018	6. 131*
420	907	0. 085	44. 785***B	513	856	-0. 018	5. 999*
420	923	0. 086	45. 768***B	513	902	-0. 018	6. 131*
420	934	-0. 001	0. 175	513	907	-0. 018	6. 131*
420	947	0. 085	44. 785***B	513	923	-0. 018	5. 999*
420	948	-0. 038	11. 190***	513	934	0. 000	0. 044
420	951	-0. 001	0. 175	513	947	-0. 018	6. 131*
420	952	-0. 001	0. 175	513	948	-0. 011	2. 844
420	954	0. 086	45. 768***B	513	951	0. 000	0. 044
420	969	-0. 001	0. 175	513	952	0. 000	0. 044
420	973	-0. 001	0. 175	513	954	-0. 018	5. 999*
420	975	0. 086	45. 768***B	513	969	0. 000	0. 044
420	977	-0. 001	0. 175	513	973	0. 000	0. 044
420	978	-0. 001	0. 175	513	975	-0. 018	5. 999*
420	982	0. 085	44. 785***B	513	977	0. 000	0. 044
420	983	0. 086	45. 768***B	513	978	0. 000	0. 044
420	990	-0. 001	0. 175	513	982	-0. 018	6. 131*
420	1074	-0. 001	0. 175	513	983	-0. 018	5. 999*
420	1078	0. 013	11. 729***	513	990	0. 000	0. 044
420	1114	0. 086	45. 768***B	513	1074	0. 000	0. 044
420	1117	0. 086	45. 768***B	513	1078	-0. 001	0. 181
420	1167	0. 085	44. 785***B	513	1114	-0. 018	5. 999*
420	1181	0. 086	45. 768***B	513	1117	-0. 018	5. 999*
420	1200	0. 086	45. 768***B	513	1167	-0. 018	6. 131*
420	1228	0. 085	44. 785***B	513	1181	-0. 018	5. 999*
420	1243	0. 086	45. 768***B	513	1200	-0. 018	5. 999*
420	1247	0. 085	44. 785***B	513	1228	-0. 018	6. 131*
420	1273	0. 085	44. 785***B	513	1243	-0. 018	5. 999*
420	1277	-0. 001	0. 175	513	1247	-0. 018	6. 131*
420	1295	0. 085	44. 785***B	513	1273	-0. 018	6. 131*
420	1334	0. 085	44. 785***B	513	1277	0. 000	0. 044
420	1337	0. 085	44. 785***B	513	1295	-0. 018	6. 131*
420	1340	0. 086	45. 768***B	513	1334	-0. 018	6. 131*
420	1346	0. 085	44. 785***B	513	1337	-0. 018	6. 131*
420	1370	-0. 001	0. 175	513	1340	-0. 018	5. 999*
420	1373	-0. 001	0. 175	513	1346	-0. 018	6. 131*
420	1378	-0. 038	11. 190***	513	1370	0. 000	0. 044
420	1383	-0. 001	0. 175	513	1373	0. 000	0. 044
420	1386	0. 086	45. 768***B	513	1378	-0. 011	2. 844
420	1398	0. 086	45. 768***B	513	1383	0. 000	0. 044
420	1433	-0. 001	0. 175	513	1386	-0. 018	5. 999*
420	1472	0. 086	45. 768***B	513	1398	-0. 018	5. 999*
420	1490	0. 086	45. 768***B	513	1433	0. 000	0. 044
420	1495	0. 086	45. 768***B	513	1472	-0. 018	5. 999*
420	1502	0. 085	44. 785***B	513	1490	-0. 018	5. 999*
513	745	-0. 018	5. 999*	513	1495	-0. 018	5. 999*
513	749	-0. 018	5. 999*	513	1502	-0. 018	6. 131*
513	757	0. 000	0. 044	513	870	-0. 018	5. 999*
513	769	0. 000	0. 044	513	872	0. 000	0. 044
513	776	-0. 018	5. 999*	513	882	-0. 018	5. 999*

513	788	-0. 018	5. 999*
513	794	-0. 017	5. 870*
540	757	0. 000	0. 005
540	769	0. 000	0. 005
540	776	0. 003	1. 400
540	788	0. 003	1. 400
540	794	0. 003	1. 431
540	798	0. 003	1. 370
540	804	0. 003	1. 400
540	814	0. 004	2. 953
540	829	0. 000	0. 005
540	838	0. 003	1. 370
540	856	0. 003	1. 400
540	870	0. 003	1. 400
540	872	0. 000	0. 005
540	882	0. 003	1. 400
540	902	0. 003	1. 370
540	907	0. 003	1. 370
540	923	0. 003	1. 400
540	934	0. 000	0. 005
540	947	0. 003	1. 370
540	948	0. 004	2. 953
540	951	0. 000	0. 005
540	952	0. 000	0. 005
540	954	0. 003	1. 400
540	969	0. 000	0. 005
540	973	0. 000	0. 005
540	975	0. 003	1. 400
540	977	0. 000	0. 005
540	978	0. 000	0. 005
540	982	0. 003	1. 370
540	983	0. 003	1. 400
540	990	0. 000	0. 005
540	1074	0. 000	0. 005
540	1078	0. 000	0. 022
540	1114	0. 003	1. 400
540	1117	0. 003	1. 400
540	1167	0. 003	1. 370
540	1181	0. 003	1. 400
540	1200	0. 003	1. 400
540	1228	0. 003	1. 370
540	1243	0. 003	1. 400
540	1247	0. 003	1. 370
540	1273	0. 003	1. 370
540	1277	0. 000	0. 005
540	1295	0. 003	1. 370
540	1334	0. 003	1. 370
540	1337	0. 003	1. 370
540	1340	0. 003	1. 400
540	1346	0. 003	1. 370
540	1370	0. 000	0. 005
540	1373	0. 000	0. 005
540	1378	0. 004	2. 953
540	1383	0. 000	0. 005
540	1386	0. 003	1. 400
540	1398	0. 003	1. 400

540	745	0. 003	1. 400
540	749	0. 003	1. 400
540	1490	0. 003	1. 400
540	1495	0. 003	1. 400
540	1502	0. 003	1. 370
564	745	0. 092	49. 654***B
564	749	0. 092	49. 654***B
564	757	-0. 001	0. 190
564	769	0. 004	5. 328*
564	776	0. 092	49. 654***B
564	788	0. 092	49. 654***B
564	794	0. 088	45. 150***B
564	798	0. 092	48. 587***B
564	804	0. 092	49. 654***B
564	814	-0. 040	12. 140***
564	829	-0. 001	0. 190
564	838	0. 092	48. 587***B
564	856	0. 092	49. 654***B
564	870	0. 092	49. 654***B
564	872	-0. 001	0. 190
564	882	0. 092	49. 654***B
564	902	0. 092	48. 587***B
564	907	0. 092	48. 587***B
564	923	0. 092	49. 654***B
564	934	-0. 001	0. 190
564	947	0. 092	48. 587***B
564	948	-0. 040	12. 140***
564	951	-0. 001	0. 190
564	952	-0. 001	0. 190
564	954	0. 092	49. 654***B
564	969	-0. 001	0. 190
564	973	-0. 001	0. 190
564	975	0. 092	49. 654***B
564	977	-0. 001	0. 190
564	978	-0. 001	0. 190
564	982	0. 092	48. 587***B
564	983	0. 092	49. 654***B
564	990	-0. 001	0. 190
564	1074	-0. 001	0. 190
564	1078	0. 013	10. 699**
564	1114	0. 092	49. 654***B
564	1117	0. 092	49. 654***B
564	1167	0. 092	48. 587***B
564	1181	0. 092	49. 654***B
564	1200	0. 092	49. 654***B
564	1228	0. 092	48. 587***B
564	1243	0. 092	49. 654***B
564	1247	0. 092	48. 587***B
564	1273	0. 092	48. 587***B
564	1277	0. 004	5. 328*
564	1295	0. 092	48. 587***B
564	1334	0. 092	48. 587***B
564	1337	0. 092	48. 587***B
564	1340	0. 092	49. 654***B
564	1346	0. 092	48. 587***B
564	1370	0. 004	5. 328*

540	1433	0. 000	0. 005	564	1373	-0. 001	0. 190
540	1472	0. 003	1. 400	564	1378	-0. 040	12. 140***
564	1383	0. 004	5. 328*	686	975	0. 243	189. 000***B
564	1386	0. 092	49. 654***B	686	977	-0. 002	0. 722
564	1398	0. 092	49. 654***B	686	978	-0. 002	0. 722
564	1433	-0. 001	0. 190	686	982	0. 241	184. 941***B
564	1472	0. 092	49. 654***B	686	983	0. 243	189. 000***B
564	1490	0. 092	49. 654***B	686	990	-0. 002	0. 722
564	1495	0. 092	49. 654***B	686	1074	-0. 002	0. 722
564	1502	0. 092	48. 587***B	686	1078	0. 012	5. 690*
686	745	0. 243	189. 000***B	686	1114	0. 243	189. 000***B
686	749	0. 243	189. 000***B	686	1117	0. 243	189. 000***B
686	757	-0. 002	0. 722	686	1167	0. 241	184. 941***B
686	769	0. 003	1. 400	686	1181	0. 243	189. 000***B
686	776	0. 243	189. 000***B	686	1200	0. 243	189. 000***B
686	788	0. 243	189. 000***B	686	1228	0. 241	184. 941***B
686	794	0. 24	184. 927***B	686	1243	0. 243	189. 000***B
686	798	0. 241	184. 941***B	686	1247	0. 241	184. 941***B
686	804	0. 243	189. 000***B	686	1273	0. 241	184. 941***B
686	814	0. 148	89. 588***B	686	1277	0. 003	1. 400
686	829	-0. 002	0. 722	686	1295	0. 241	184. 941***B
686	838	0. 241	184. 941***B	686	1334	0. 241	184. 941***B
686	856	0. 243	189. 000***B	686	1337	0. 241	184. 941***B
686	870	0. 243	189. 000***B	686	1340	0. 243	189. 000***B
686	872	-0. 002	0. 722	686	1346	0. 241	184. 941***B
686	882	0. 243	189. 000***B	686	1370	0. 003	1. 400
686	902	0. 241	184. 941***B	686	1373	-0. 002	0. 722
686	907	0. 241	184. 941***B	686	1378	0. 148	89. 588***B
686	923	0. 243	189. 000***B	686	1383	0. 003	1. 400
686	934	-0. 002	0. 722	686	1386	0. 243	189. 000***B
686	947	0. 241	184. 941***B	686	1398	0. 243	189. 000***B
686	948	0. 148	89. 588***B	686	1433	-0. 002	0. 722
686	951	-0. 002	0. 722	686	1472	0. 243	189. 000***B
686	952	-0. 002	0. 722	686	1490	0. 243	189. 000***B
686	954	0. 243	189. 000***B	686	1495	0. 243	189. 000***B
686	969	-0. 002	0. 722	686	1502	0. 241	184. 941***B
686	973	-0. 002	0. 722				

Values of the *D* parameter.
The significant disequilibrium linkage after Bonferroni’s correction is indicated by the letter B.
* $p < 0.005$; ** $p < 0.010$; *** $p < 0.001$
